# Secondary Electron Hyperspectral Imaging of Carbons: New Insights and Good Practice Guide

**DOI:** 10.1002/advs.202501907

**Published:** 2025-06-17

**Authors:** James F. Nohl, Nicholas T.H. Farr, Maria Rosaria Acocella, Alexander J. Knight, Gareth M. Hughes, Jingqiong Zhang, Stuart Robertson, Stuart Micklethwaite, Sean Murphy, Tereza Motlová, Christopher Walker, Alexander I. Tartakovskii, Filip Mika, Zuzana Pokorná, Steve Tear, Andrew Pratt, Nancy L. Ford, Nicole Hondow, Mark A. E. Jepson, Lyudmila S. Mihaylova, Nik Reeves‐McLaren, Serena A. Cussen, Cornelia Rodenburg

**Affiliations:** ^1^ School of Chemical Materials and Biological Engineering The University of Sheffield Mappin Street Sheffield S1 3JD UK; ^2^ The Faraday Institution Quad One Harwell Campus Becquerel Avenue Didcot OX11 0RA UK; ^3^ Insigneo Institute for in Silico Medicine Pam Liversidge Building Sir Robert Hadfield Building The University of Sheffield Mappin Street Sheffield S1 3JD UK; ^4^ Department of Chemistry and Biology “A. Zambelli” University of Salerno Via Giovanni Paolo II, 132, Fisciano Salerno 84084 Italy; ^5^ School of Mathematical and Physical Sciences The University of Sheffield Sheffield S3 7RH UK; ^6^ David Cockayne Centre for Electron Microscopy Department of Materials University of Oxford Parks Road Oxford OX1 3PH UK; ^7^ School of Electrical and Electronic Engineering The University of Sheffield Amy Johnson Building Portobello Street Sheffield S1 3JD UK; ^8^ Loughborough Materials Characterisation Centre Loughborough University Epinal Way Loughborough Leicestershire LE11 3TU UK; ^9^ School of Chemical and Process Engineering University of Leeds Leeds LS2 9JT UK; ^10^ Department of Oral Biological and Medical Sciences University of British Columbia Vancouver V6T 1Z3 Canada; ^11^ Institute of Scientific Instruments of the CAS Královopolská 147 Brno‐Královo Pole 612 00 Czech Republic; ^12^ Department of Microelectronics Brno University of Technology Technická 3058/10 Brno 61600 Czech Republic; ^13^ School of Physics Engineering and Technology University of York, Heslington York YO10 5DD UK; ^14^ Department of Materials Loughborough University Epinal Way Loughborough Leicestershire LE11 3TU UK; ^15^ School of Chemistry University College Dublin Belfield Dublin 4 Ireland

**Keywords:** functional groups, graphitic carbon, secondary electron hyperspectral imaging, surface chemistry, surface spectroscopy

## Abstract

Energy storage technologies such as lithium‐ion batteries (LIBs) incorporate carbon components key to their function. Graphite and carbon binder components in LIB electrodes are engineered to deliver critical electrical and mechanical properties, as are the surface chemistry and morphology of carbon blacks (CBs) in LIBs and catalysts. The challenge of relating surface chemistry to morphology is complicated by the numerous forms of carbon bonding and potential for surface functional groups. Furthermore, materials processing can influence bonding and structure of carbon at multiple length scales, as seen in mechanochemical functionalization of CBs. To understand the nature of carbon surfaces, secondary electron hyperspectral imaging (SEHI) is introduced as a spatially resolved analysis bridging the nano to microscale. The ability to provide novel insights is demonstrated three example applications: observation of nanoscale “satellite” particles of amorphous hydrogenated carbon on graphitic CB particles, differentiation between graphitic and amorphous hydrogenated nano‐thickness carbon coatings on particles of lithium iron phosphate, and differentiation between graphitic carbon active material and carbon binder domain in a LIB anode material. SEHI analysis using peak fitting models for graphitic and disordered carbons is developed based on reference materials and standard spectroscopic methods: Raman spectroscopy and X‐ray photoelectron spectroscopy.

## Graphical abstract

Secondary hyperspectral imaging of carbon surfaces is assessed with reference materials and in comparison to standard spectroscopies. Characterization of functional carbon materials from battery research reveals otherwise inaccessible information about the distribution of sp^2^ and amorphous carbon, as shown in graphite anode and carbon coated lithium iron phosphate. Surface functional groups on carbon black are mapped down to the nanoscale.

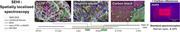

## 1 Introduction

Lithium‐ion battery (LIB) electrodes require functional carbon components to optimize electrochemical performance. Conventional LIB lithium‐ion battery anodes, for example, are composed of graphitic and polymeric carbon distributed to provide electronic, ionic and mechanical function. These thin‐film electrodes comprise of nanoscale carbon blacks (CBs), fibers, and macroscale particles of graphite, within a polymer binder domain. In addition to the beneficial transport properties, morphological properties such as porosity and surface roughness also have impact on the electrode performance.^[^ ^1^ ^]^ In the case of the cathode, ultrathin carbon coatings are typically applied to the surface of lithium iron phosphate (LFP) cathode particles to introduce electronically conductive graphitic character, although some disorder is common.^[^ ^2^ ^]^

There are several methods by which carbon can be applied as a coating or an additive for Li ion battery electrodes. Mechanochemistry by ball milling is a solvent free and low‐cost method to modify materials size and surface functional groups, for instance to improve the electrochemical properties of carbon materials which are important to a range of energy applications including cheaper, higher performance batteries.^[^ ^3^ ^]^ Challenges in ball milling are: that homogeneity is difficult to achieve, and that both size distribution and surface properties need to be optimized in one step.^[^ ^4^, ^5^, ^6^ ^]^ During ball milling of carbons, disorder can be reduced or increased (depending on the starting material) and new surface functional groups can be introduced.^[^ ^7^ ^]^ Thus, to optimize or scale‐up ball milling of carbons, morphological features like particle size distribution, porosity, and roughness should be related to carbon character and surface functional groups, ideally over length scales which relate to powders (microscale) down to the smallest primary particles (nanoscale). Scanning electron microscopy (SEM) is already widely used to analyze morphological properties but does not deliver chemical information about the nature of the carbon which spectroscopy could deliver.

The variety of chemistries and morphologies in application materials produced through materials engineering and processing presents a characterization challenge for existing spatially resolved spectroscopy techniques. The information they provide may not extend to the upper and lower length scales of features in the materials.

**Figure 1:**
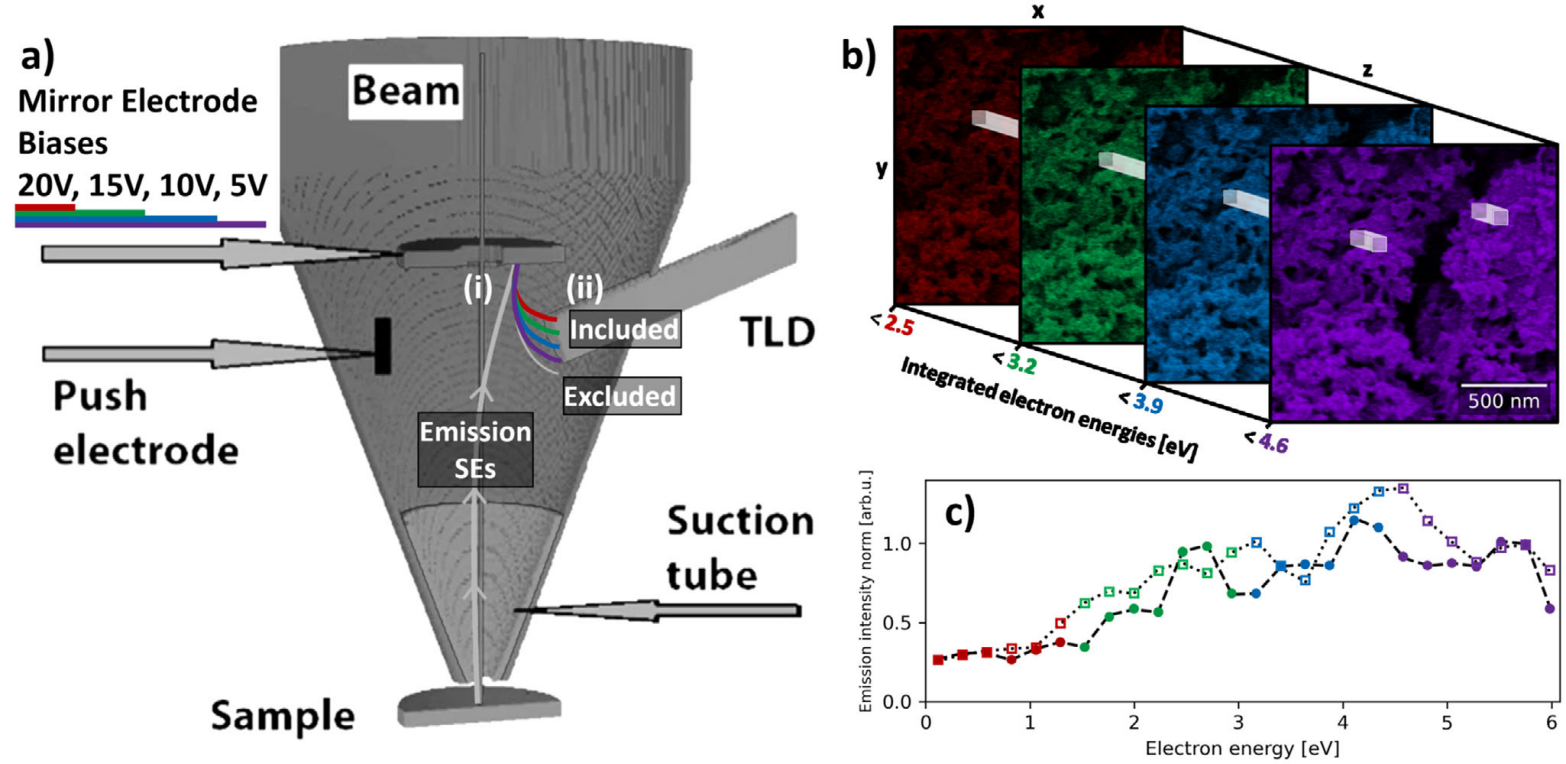
a) Schematic of Helios Elstar column from Konvalina et al., Materials (2019). Adapted under the terms of the CC BY 4.0 license,^[^ ^16^ ^]^ where emission electron trajectories are accelerated by the suction tube bias, i) deflected by the mirror electrode which is stepped through bias voltages, then ii) excluded from or included in detection by the through lens detector (TLD). b) The SEHI data volume is made up of SEs that pass the low‐pass filter applied by the mirror electrode, from Nohl et al., Micron (2022). Adapted under the terms of the CC BY 4.0 license.^[^ ^13^ ^]^ c) The SEHI data volume (from a LIB cathode material) is differentiated with respect to the energy (*z*‐dimension) to yield SE spectra. Each point in the series corresponds to a mirror electrode step. SE spectra can be derived from regions of the SEHI data volume to compare SE spectra between regions, as shown by the dashed and dotted lines in (c).

One technique which can fill this gap is secondary electron hyperspectral imaging (SEHI). SEHI is a low‐voltage SEM (LV‐SEM) technique which yields spatially localized surface chemical information about functional materials.^[^ ^8^, ^9^, ^10^, ^11^, ^12^, ^13^ ^]^ For carbon, low beam acceleration voltage is necessary for Secondary electron (SE) spectroscopy, as the SE yield is ≈0.6 and total electron yield is near one at 1 keV incident electron energy.^[^ ^14^, ^15^ ^]^ SE spectra of highly oriented pyrolytic graphite (HOPG) produced by a Bessel‐box add‐on detector showed an increase in the SE spectrum peak intensity as the beam accelerating voltage was decreased from 10 to 1 kV (Figure S1a, Supporting Information). Without the need for an add‐on spectrometer, SE spectra can be derived from SEHI data volumes. In SEHI, the mirror electrode in an Elstar column (Thermofisher/FEI) is used as a low pass filter to image an area sequentially with increasing energy cutoff (**Figure** **1**a). The resulting data volume is differentiated in the energy axis (*z*‐axis in Figure 1b) to produce a hyperspectral image. Each pixel in the image has an associated SE spectrum in the *z*‐dimension. SE spectra from areas of interest can be plotted as in Figure 1c.

**Figure 2:**
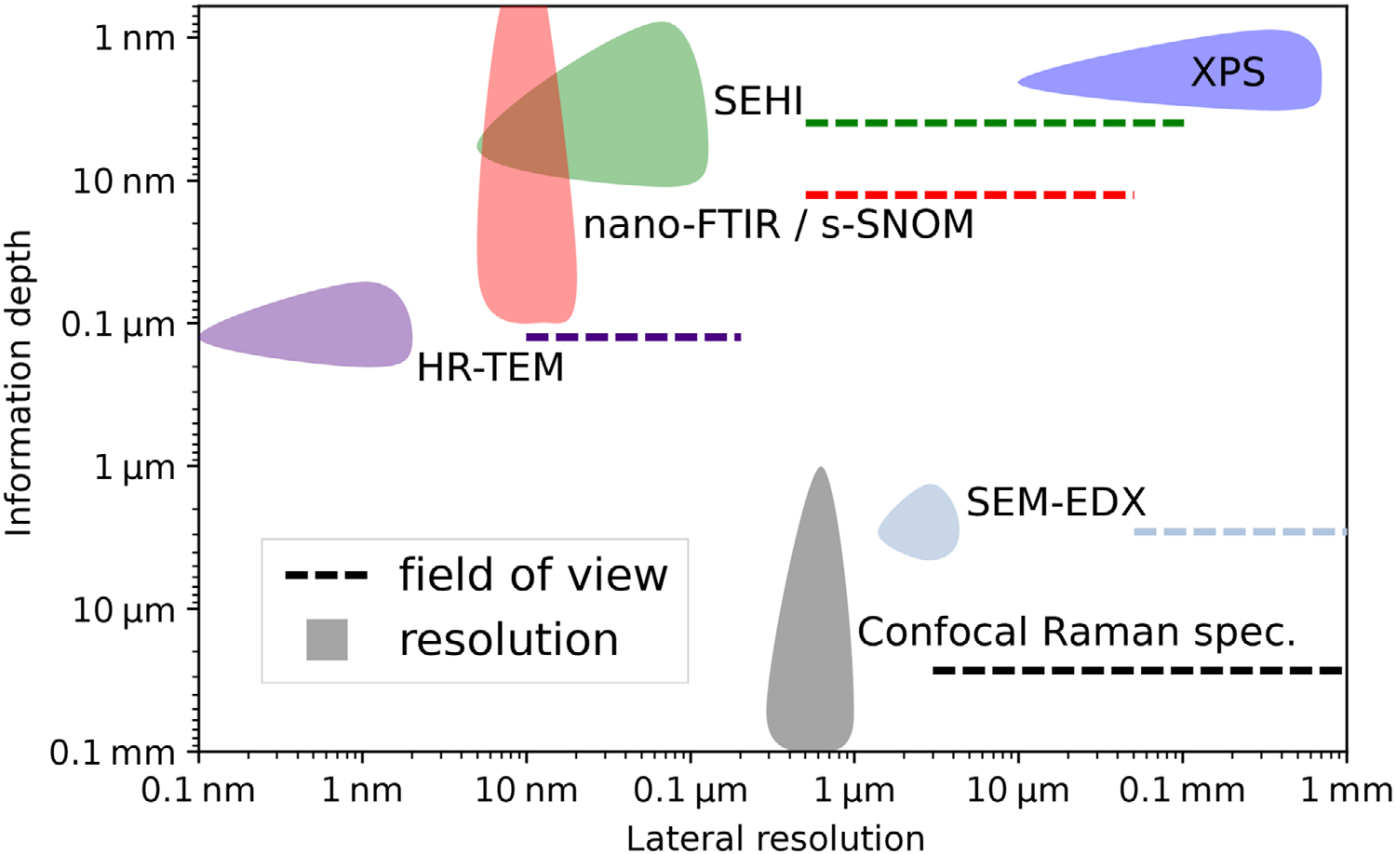
Plot of the spatial localization of information from characterization techniques: SEHI, confocal Raman spectroscopy, X‐ray photoelectron spectroscopy (XPS), nano‐Fourier transformed infrared spectroscopy (FTIR)/scattering scanning nearfield optical microscopy (s‐SNOM), SEM ‐energy dispersive X‐ray analysis (EDX), and high‐resolution transmission electron microscopy (HR‐TEM). Shaded regions represent resolution and information depth. Horizontal dashed lines represent the range of horizontal field widths (HFW) accessible, i.e., for SEHI the HFW range is 500 nm to 0.1 mm (lower limit: 100× minimum lateral resolution, upper limit: TLD HFW).

By plotting the spatial localization of signal from spectroscopic characterization techniques found in literature and in this study, we find SEHI uniquely placed to give information with spatial resolution as low as 5 nm (as measured in 5.1.2) over a horizontal field width (HFW) from 500 nm to 100 µm, and a depth resolution between 0.77 and 11 nm (**Figure** **2** and **Table** **1**).

**Table 1:** Accompanying (Figure 2), table of technique spatial localization, and irradiation and signal types. KE is kinetic energy.

Technique	Information localization		Irradiation/signal
	XY	Z	
SEHI	5 nm (electron spot and mean escape depth)	0.77–11 nm (mean escape depth)^[^ ^17^, ^18^ ^]^	Incident electron (1 keV KE)/Secondary electrons (<10 eV KE)
			
Confocal Raman spectroscopy	290 nm (Rayleigh criterion)^[^ ^19^ ^]^	1 µm (focal length)^[^ ^20^ ^]^	Laser (514 nm)/Raman scattered light
			
XPS	10 µm (X‐ray spot and analyzer lens)^[^ ^6^, ^7^ ^]^	0.9–3.3 nm (3× inelastic mean free path)^[^ ^21^ ^]^	X‐ray (1486.6 eV Al‐Kα)/Photoelectrons (<1486 eV KE)
Nano‐FTIR/s‐SNOM	10 nm (tip apex)^[^ ^22^, ^23^ ^]^	0–100 nm (near‐field penetration depth)^[^ ^24^, ^25^ ^]^	IR (2.5–25 um)/Backscattered light absorbance^[^ ^26^ ^]^
SEM‐EDX	1.4–4.3 µm (X‐ray excitation region)^[^ ^27^ ^]^	1.4–4.3 µm (X‐ray excitation region)^[^ ^27^ ^]^	Incident electron (10–20 keV KE)/X‐rays (C Kα 277 eV)^[^ ^28^ ^]^
			
HR‐TEM	0.1 nm^[^ ^29^, ^30^, ^31^ ^]^	50–200 nm (prepared sample thickness)	Incident electron (30–300 keV KE)/transmitted and backscattered electrons
SEM‐STEM EDX	10 nm^[^ ^32^, ^33^ ^]^	10 nm (prepared sample thickness)^[^ ^32^, ^33^ ^]^	Incident electron (20–30 keV KE)/X‐rays^[^ ^32^, ^33^ ^]^

**Figure 3:**
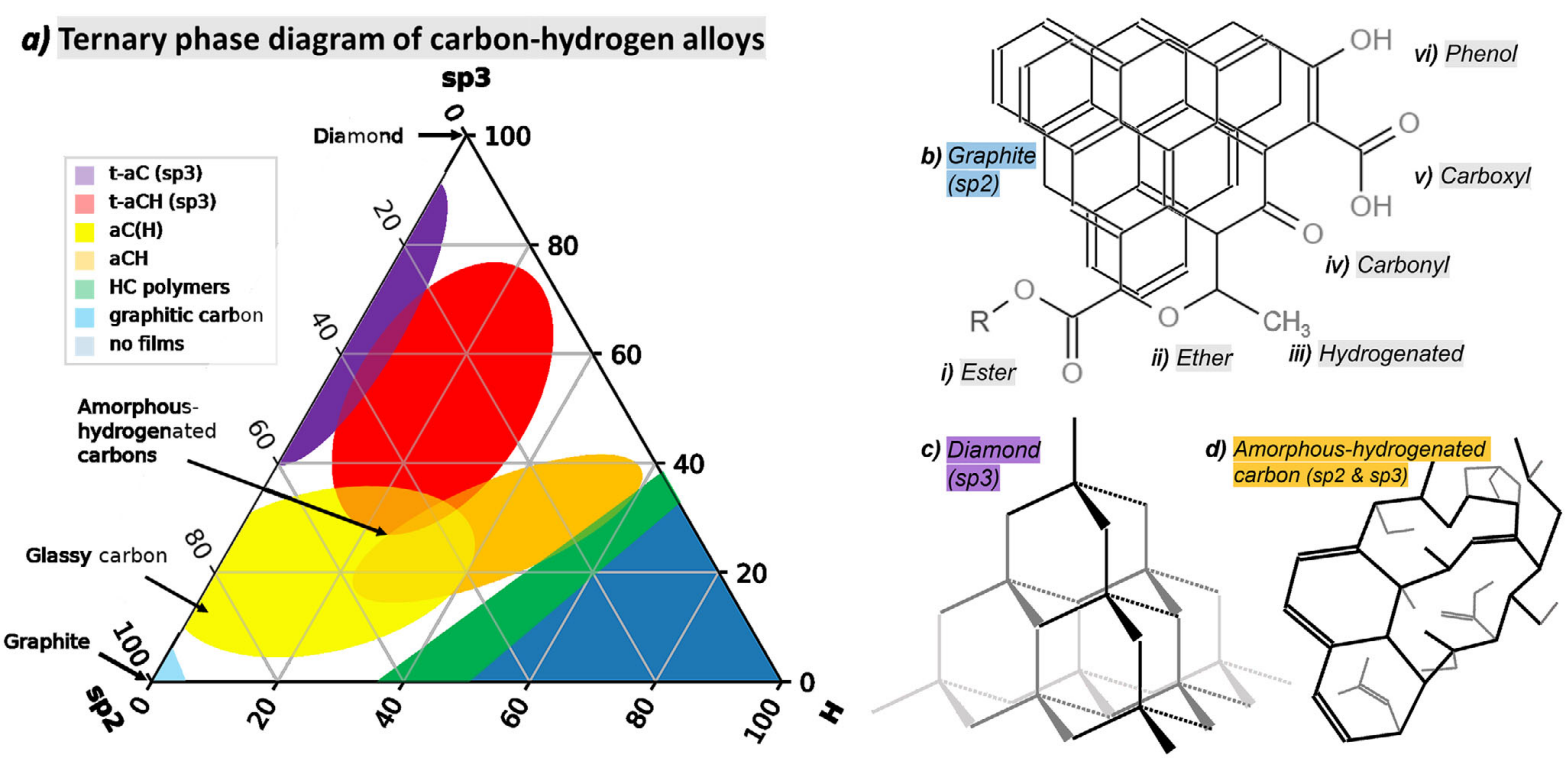
a) Ternary phase diagram of carbon–hydrogen alloys showing composition domain regions for tetrahedral (sp^3^ bonded) amorphous carbon (t‐aC) and amorphous‐hydrogenated carbon (t‐aCH), sp^2^ bonded amorphous hydrogenated carbons (aCH; aC(H)), hydrocarbon (HC) polymers, graphitic carbon and hydrocarbons that do not form films. Regions informed by refs. [38, 44, 45]. b) Structures (drawn using JME molecular editor) of graphite (sp^2^ bonded carbon) and oxygen and hydrogen containing functional groups: i) ester (COOR), ii) ether (C‐O‐R), iii) hydrogenated carbon (CH_3_), iv) carbonyl (CO), v) carboxyl (COOH), vi) phenol (OH). Structures of c) diamond (sp^3^ bonded carbon) and d) amorphous‐hydrogenated carbon (sp^2^ and sp^3^ bonded).

The ternary phase diagram of carbon‐hydrogen alloys (**Figure** **3**a) represents the carbon‐hydrogen phase system in which many functional carbon materials exist, such as CB feedstocks, carbon coatings on lithium iron phosphate (LFP), or graphitic components in the LIB anode. The axis between graphite (with sp^2^‐hybridized carbon bonding, Figure 3b) and diamond (with sp^3^ bonding, Figure 3c) indicates a continuum of disordered carbon with increasing tetrahedral and sp^3^ bonding character (represented in the schematic in Figure 3d). Such systems have been extensively studied by Raman spectroscopy, as spectra are sensitive to graphitic and diamond‐like carbon bond vibrations in resonant modes.^[^ ^34^, ^35^, ^36^, ^37^, ^38^, ^39^, ^40^, ^41^ ^]^

The surfaces of carbon materials can form functional groups between air and reactive carbon bonds, for example hanging bonds on the edges of planes of graphite,^[^ ^42^ ^]^ shown in Figure 3b‐i–vi. Applications of CBs in carbon capture and waste‐water treatment result from properties related to surface functionalization. X‐ray photoelectron spectroscopy (XPS) is often used to characterize the surface chemistry of these materials, for example returning information about the O/C ratio of functional groups at the surface.^[^ ^43^ ^]^

Given the importance of information derived from the surface of materials, carbon analysis by SEHI requires special care due to the potential for carbonaceous contamination deposition during analysis. There is a growing body of work concerned with electron beam induced deposition (EBID) contamination of residual hydrocarbons in electron microscopes including focused ion beam (FIB‐)SEMs and in LV‐SEM operation modes (<5 keV).^[^ ^46^, ^47^ ^]^ Resulting models related the deposition of contaminant molecules to variables such as chamber pressure, electron energy and dose, and contaminant cracking and deposition cross‐sections. SEHI monitored EBID contamination in situ, but experiments relating SE spectrum evolution to these variables could prove valuable in parameterizing the SEHI experimental space and EBID contamination in LV‐SEM more generally.^[^ ^42^ ^]^

Where there is a mixed surface of hydrogenated, functionalized, and contaminated carbons, the work function can dominate the contributions to SE spectral peaks.^[^ ^42^ ^]^ However, fine structures in SE spectra of sp^2^‐hybridised carbons, diamond‐like carbons, and polymers are not adequately described by the work function‐dominated model of SE spectra (first developed by Chung and Everhart^[^ ^51^ ^]^).^[^ ^48^, ^49^, ^50^ ^]^ Understanding of fine structures in SE spectra has recently been developed by comparison to bulk valence band density of states models.^[^ ^52^ ^]^ However, such models do not exist for disordered materials such as disordered and amorphous carbons where coordination of carbon is varied over multiple length scales and there are surface effects such as functional groups. Meanwhile, interpretation of experimental data remains challenging due to varied characteristics of analyzer systems,^[^ ^48^, ^53^ ^]^ and the influence of specimen surface preparation and treatment,^[^ ^52^ ^]^ beam voltage, and background and interaction depth effects.^[^ ^54^ ^]^

The following study addresses the challenges identified to gain insight into the surface chemistry of carbons. These are:

- How can SE spectrum data collected by SEHI be analyzed quantitatively?
- How can EBID contamination be measured and minimized?
- What length scales are accessible to SEHI with SE spectrum fitting?
- How can carbon disorder and oxygen functional groups be included in SE spectrum models and mapped with SEHI?

## 2 Outline of Experiments

**Figure 4:**
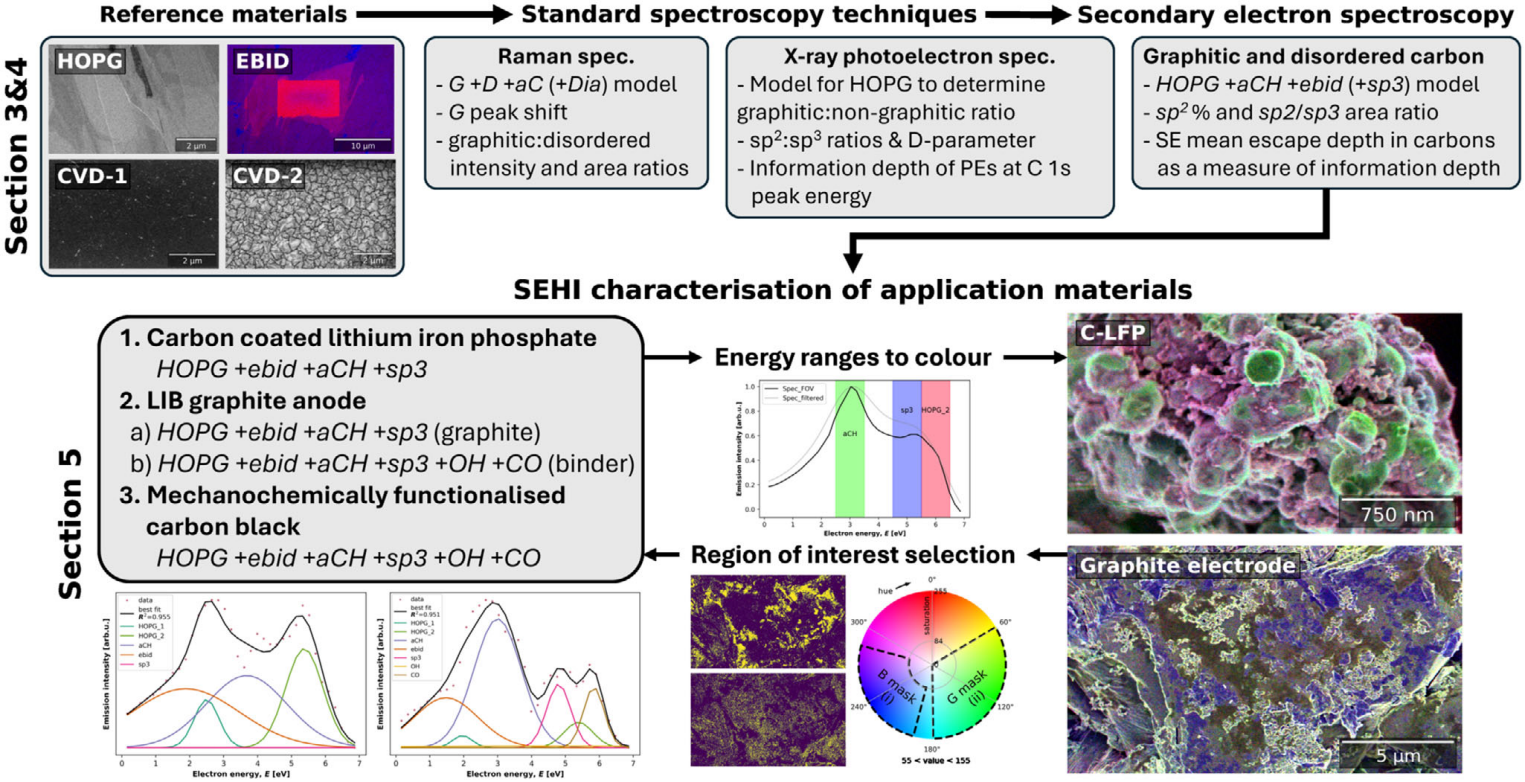
Schematic of experimental design. Sections 3 and 4: three reference materials, highly oriented pyrolytic graphite (HOPG), and two chemical vapor deposited (CVD) carbons were measured by standard spectroscopic techniques Raman spectroscopy and XPS. Standard spectroscopy fitting models used to characterize reference materials informed the development of fitting models for SE spectroscopy. Section 5: the SE spectrum fitting models are shown for each of the application materials. The analysis workflow includes coloring SEHI images in energy ranges and color thresholding to produce mask regions for further SE spectrum fitting.

The study is designed to address the challenges outlined above. The workflow in each section of the study is presented in **Figure** **4**. In Section 3, we measure Raman and X‐ray photoelectron spectra of reference materials from graphite to disordered carbon and compare these to SE spectra to build fitting models for SE spectra of graphitic and disordered carbon materials (challenge i). In Section 4, we calibrate energy filtering for SEHI in five FIB‐SEMs (Xe^+^ and Ga^+^ FIBs), and investigate the effect of imaging parameters on EBID (challenge ii). In Section 5, we use SEHI to characterize example application materials with carbon components, relating morphology to surface chemistry features ranging from nano to macroscale (challenge iii). Two of these application materials contain carbon–oxygen groups, for which SE spectrum fitting models were developed (challenge iv).

## 3 Comparison of Standard Spectroscopic Techniques to Secondary Electron Hyperspectral Imaging (SEHI)

### 3.1 Raman Spectroscopy and X‐ray Photoelectron Spectroscopy (XPS) of Reference Materials

Raman spectroscopy and XPS are spectroscopic techniques which are sensitive to local bonding, order and electronic structure.^[^ ^37^, ^55^ ^]^ Raman spectroscopy and XPS are widely used to gain understanding of carbon material surfaces and sub‐surfaces (see the lateral and depth resolution of techniques for analysis of carbon materials in Figure 2). Raman spectroscopy has been used extensively to characterize graphitic and amorphous carbons, as well as diamond‐like films. As such, there is extensive literature to aid collection and interpretation of Raman spectra to gain insights into the carbon character.^[^ ^34^, ^35^, ^36^, ^37^, ^38^, ^39^, ^40^, ^41^ ^]^

HOPG and two disordered carbon films prepared by chemical vapor deposition (CVD) (CVD‐1) from ref. [56] and CVD‐2 from NeoCoat SA, Switzerland were used as reference materials. These thin films were chosen based on previously published *I*_D_/*I*_G_ ratios and XPS data in ref. [53]. For CVD‐1 (*I*_D_/*I*_G_ = 0.46 ± 0.02), XPS indicated the presence of O and N also typically found in EBID. Thus, the HOPG fitting model should be applicable. Furthermore, CVD‐2 (*I*_D_/*I*_G_ = 0.48 ± 0.02) has ID/IG ratio but exhibits clearly visible microstructure and represents a commercially available material (NeoCoat SA, Switzerland). Despite the similar *I*_D_/*I*_G_ ratios the CVD‐1 and CVD‐2 differ in Raman peak shape substantially.

**Figure 5:**
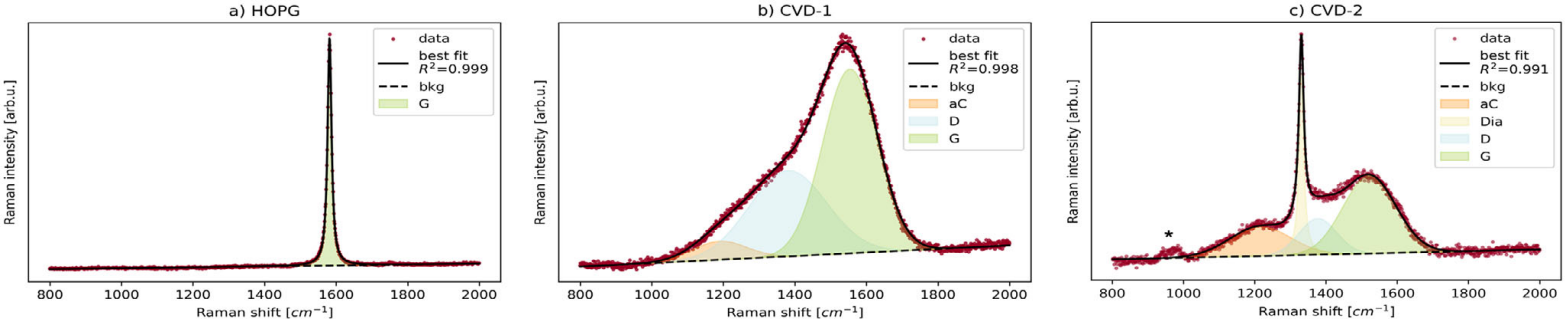
a–c) Raman spectra in the region 800 to 2000 cm^−1^ of highly oriented pyrolytic graphite (HOPG), CVD‐1 and CVD‐2. Linear background subtraction and Lorentzian peak fitted for graphitic carbon “G,” (a) Gaussian peaks for amorphous carbon “aC,” disordered carbon “D” and graphitic carbon “G,” (b, c) Lorentzian peak for diamond “Dia” (c) and “*” denotes contribution from the silicon substrate. For absolute intensity plots please see Section S2.1 and Figure S2b–d (Supporting Information).

**Figure** **5** shows Raman spectra of the three reference materials with fitting models. Experimental details are found in Section S2.1 (Supporting Information). The fitting models used were one component for the HOPG graphitic carbon *G* peak, three components for CVD‐1 amorphous carbon, disordered carbon and graphitic carbon peaks, labelled *aC*, *D*, and *G* respectively, and four components for CVD‐2 which was the CVD‐1 model plus a diamond *Dia* component.

The *G* peak centered at 1582 ± 0.1 cm^−1^ in the Raman spectrum of HOPG (Figure 5a) results from hybridized sp^2^ π bonding mode vibrations in graphitic carbon.^[^ ^38^ ^]^ This peak was labelled *G* for graphite and was fitted with a Lorentzian peak (Figure 5a) due to finite lifetime broadening of the phonon relaxation, which indicates high crystallinity of graphitic regions.^[^ ^38^ ^]^ The *G* peaks in Raman spectra of CVD‐1 and CVD‐2 (Figure 5b,c) were fitted with Gaussian curves which represent a wider distribution of phonon lifetimes in more disordered graphitic regions of the material.^[^ ^38^ ^]^ The full width at half maxima (FWHMs) of *G* peak fits in Figure 5a–c are 16 ± 0.1, 177 ± 0.9, and 174 ± 1.8 cm^−1^. The HOPG *G* peak FWHM is ≈10× lower than that of the *G* peaks in spectra of CVD‐1 and CVD‐2. The similarity in CVD‐1 and CVD‐2 carbon *G* peak FWHMs indicate that the disorder of graphitic regions in the two references is similar.

There are various spectral qualities that provide information about non‐graphitic carbon. Ferrari and Robertson observed shifts in the *G* peak center along a pathway of increasing disorder from i) graphite (1581 cm^−1^) with introduced disorder to ii) nano‐crystalline graphite (1600 cm^−1^), with amorphization to iii) disordered graphitic carbon, aC (1510 cm^−1^), and finally iv) tetrahedral amorphous carbon, t‐aC with a *G* peak center increase to 1570 cm^−1^.^[^ ^38^ ^]^ Through the spectra in Figure 5a–c, the *G* peak center shifts from 1582 ± 0.1 cm^−1^ (HOPG) to 1553 ± 0.3 cm^−1^ (CVD‐1) to 1522 ± 1.0 cm^−1^ (CVD‐2) indicating samples with *G* peak centers typical of aC (CVD‐1) and t‐aC with a proportion of sp^3^ carbon (CVD‐2).

The *D* peak is related to an A_1g_ vibration mode which can be described as a “breathing” of carbon rings which are associated with sp^3^ bonded carbon.^[^ ^36^ ^]^ The *D* peak only becomes visible in the Raman spectrum when this ring structure contains defects. While this is not a measure of sp^3^ bonding, sp^3^ defects can contribute to the defect induced disorder, and therefore the *D* peak. This is because the presence of sp^3^ bonds disrupts the sp^2^ network, leading to a prominent *D* peak. Hence the D‐peak is not visible in HOPG but appears at 1376 ± 0.0 cm^−1^ (center obtained from peak fit of CVD‐1 and CVD‐2 Raman spectra fits, see **Table** **2**). This is indicative of the presence of some sp^3^ bonded carbon.

**Table 2:** Values from Raman spectroscopy of reference materials HOPG, CVD‐1, CVD‐2. *D* peak area, *A*_D_; A peak area, *A*_G_; *D* peak intensity, *I*_D_; *G* peak intensity, *I*_G_; A peak area of amorphous carbon component, *AaC*.

Material	Raman						
	Centre (D) [cm^−1^]	FWHM (D) [cm^−1^]	Centre (G) [cm^−1^]	FWHM (G) [cm^−1^]	$\frac{A_{D}}{A_{G}}$	$\frac{\left(A_{\mathrm{aC}}+A_{D}\right)}{A_{G}}$	$\frac{I_{D}}{I_{G}}$
HOPG	–	–	1582 ± 0.1	16 ± 0.1	–	–	–
CVD‐1	1376 ± 0.0	235 ± 0.0	1553 ± 0.3	177 ± 0.9	0.60 ± 0.02	0.69 ± 0.02	0.46 ± 0.02
CVD‐2	1376 ± 0.0	130 ± 5.1	1522 ± 1.0	174 ± 1.8	0.35 ± 0.02	0.75 ± 0.05	0.48 ± 0.02

The CVD‐1 and CVD‐2 spectra were not fitted with the conventional *D* and *G* peak model^[^ ^34^, ^35^ ^]^ as in both cases, an additional Gaussian component labelled *aC* was fitted. The *aC* peak centers are 1188 ± 1.4 and 1213 ± 2.6 cm^−1^ for CVD‐1 and CVD‐2 respectively (Figure 5b,c). This relates to disordered sp^3^ bonded carbon, as observed in a broad peak at ≈1100 cm^−1^ by Ferrari and Robertson using an ultraviolet radiation excitation source (giving resonant t‐aC mode vibrations).^[^ ^39^ ^]^ Measurements using a visible light excitation source often fit only *D* and *G* components, as the component around 1100 cm^−1^ is of relatively low intensity.^[^ ^38^ ^]^

*D* and *G* peak areas and intensities were used to calculate *D*:*G* ratios to give a metric for the graphitic character of the material. The lower the *D*:*G* ratio, the fewer defects there are in the graphitic material. The intensity ratios, *I*_D_ */I*_G_, were 0.46 ± 0.02 and 0.48 ± 0.02 for CVD‐1 and CVD‐2 respectively. The area ratios, *A*_D_ */A*_G_, were 0.60 ± 0.02 and 0.35 ± 0.02 for CVD‐1 and CVD‐2 respectively. The intensity ratio indicates a similar graphitic character whereas the area ratio indicates that the CVD‐1 carbon has a higher proportion of defects in graphite domains than CVD‐2. With the inclusion of the *aC* peak in the area ratio (*A*_aC_ *+A*_D_)*/A*_G_ the ratios for CVD‐1 and CVD‐2 are the same, indicating a similar level of amorphous to graphitic carbon, with more sp^3^ and t‐aC disorder in the CVD‐2 compared to CVD‐1.

The Raman spectrum of CVD‐2 (Figure 5c) has a sharp peak fitted with a Lorentzian curve at 1331 ± 0.0 cm^−1^. This was attributed to the diamond T_2g_ vibration mode,^[^ ^40^ ^]^ previously observed at 1332–1338 cm^−1^ in spectra of diamond thin films on Si substrates.^[^ ^41^ ^]^

**Figure 6:**
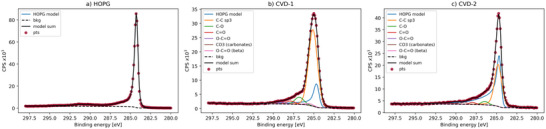
XPS spectra in the C1s peak region 298 to 280 eV from HOPG, CVD‐1 and CVD‐2 respectively. Fitted with Shirley background subtraction and components: a) two main HOPG components and two loss features (π–π* transition and shake‐up), b,c) empirical HOPG lineshape, non‐graphitic C─C, C─O, or C─N, C ═ O, or C─F, O–C ═ O, carbonates, and beta‐shifted O–C ═ O.

**Figure** **6** shows XPS spectra of the three reference materials in the C 1s region 298–280 eV with fitting models. Acquisition details are found in Section S2.2 (Supporting Information).

Briefly, the C 1s spectrum of the HOPG sample was peak‐fitted using four components to obtain an empirical lineshape for the asymmetric graphitic component: two main HOPG components and two loss features (π–π* transition and shake‐up). A shake‐up peak is a satellite peak at higher binding energy caused by secondary excitations of valence band electrons. The fitted lineshape of the C 1s spectrum of HOPG is assumed to represent 100% graphitic carbon. The C 1s spectra of CVD‐1 and CVD‐2 were fitted using the empirical HOPG lineshape and six additional components (Figure 6b,c). The C 1s peak fitting model that was used is detailed in ref. [57].

CVD‐1 and CVD‐2 fits yielded graphitic:non‐graphitic (sp^2^/sp^3^, **Table** **3**) ratios of 0.25 and 1.14 respectively, and sp^2^ contents (*Area*_HOPG_model_/*Area*_C1 s_, Table 3) of 20.23% and 53.20% respectively. XPS analysis shows there is a higher sp^3^ carbon content in the CVD‐1 film (lower sp^2^% and graphitic:non‐graphitic ratio). This seems counter to the Raman spectroscopy finding, where a diamond (sp^3^) component was found only for the CVD‐2 carbon film and the (*A*_aC_ *+A*_D_)*/A*_G_ and *I*_D_ */I*_G_ ratios (where the D peak is non‐graphitic disordered carbon bonding) were similar for the carbon films (Table 2).

**Table 3:** Values from XPS of reference materials HOPG, CVD‐1, CVD‐2.

Material	XPS		
	D‐param.	$\frac{sp^{2}}{C\;1s}$	$\frac{sp^{2}}{sp^{3}}$
HOPG	23.9 ± 0.2	100.00	–
CVD‐1	15.5 ± 0.1	20.23 ± 1.02	0.25 ± 0.02
CVD‐2	18.4 ± 0.4	53.20 ± 1.14	1.14 ± 0.05

Consideration of the information depth of the two techniques could explain the difference. The photoelectron signal decays exponentially with depth. The information depth of XPS is typically quoted as three times the inelastic mean free path of primary electrons in the specimen. 95% of photoelectron signal is emitted from within this depth. For 285 eV primary electrons, the inelastic mean free paths were calculated to be 0.9 nm in graphite and diamond and 1.1 nm in glassy carbon,^[^ ^21^ ^]^ giving information depths of 2.7 and 3.3 nm respectively. In defect free graphene this equates to the first ≈8 graphene layers in graphite (where the *c*‐axis distance between graphene layers is 0.3347 nm).^[^ ^58^ ^]^ Confocal Raman spectroscopy probes a depth of at least 1 µm which,^[^ ^20^ ^]^ in the case of the CVD carbon films, includes the whole sample depth. In fact, the Raman spectrum of CVD‐2 includes emission peaks from the silicon wafer substrate marked by “*” in Figure 5c (the Raman spectrum of silicon is shown in Figure S2a, Supporting Information). Thus, the depth sensitivity of the two techniques, which is different by an order >1000, gives sub‐surface (Raman spectroscopy) and surface (XPS) characterization of the sample. Consideration of the surface sensitivity of the two standard spectroscopic techniques is therefore an important factor when comparing to the SE spectra of these materials produced by SEHI. The difference in information depth is most likely responsible for the different outcomes of XPS and Raman peak fitting analysis. For films similar to CVD‐1 there have been reports that the surface differs from the bulk of the coating as observed by comparing XPS and ellipsometry studies.^[^ ^59^ ^]^ Therefore, Raman spectroscopy was mainly used to inform what components would need to be included as a minimum in the SEHI fitting model. As a result, we included an amorphous carbon component and sp^3^ component in the fitting model for CVD‐1 and CVD‐2 in addition to the HOPG model peaks.

### 3.2 SEHI of Reference Materials

**Figure 7:**
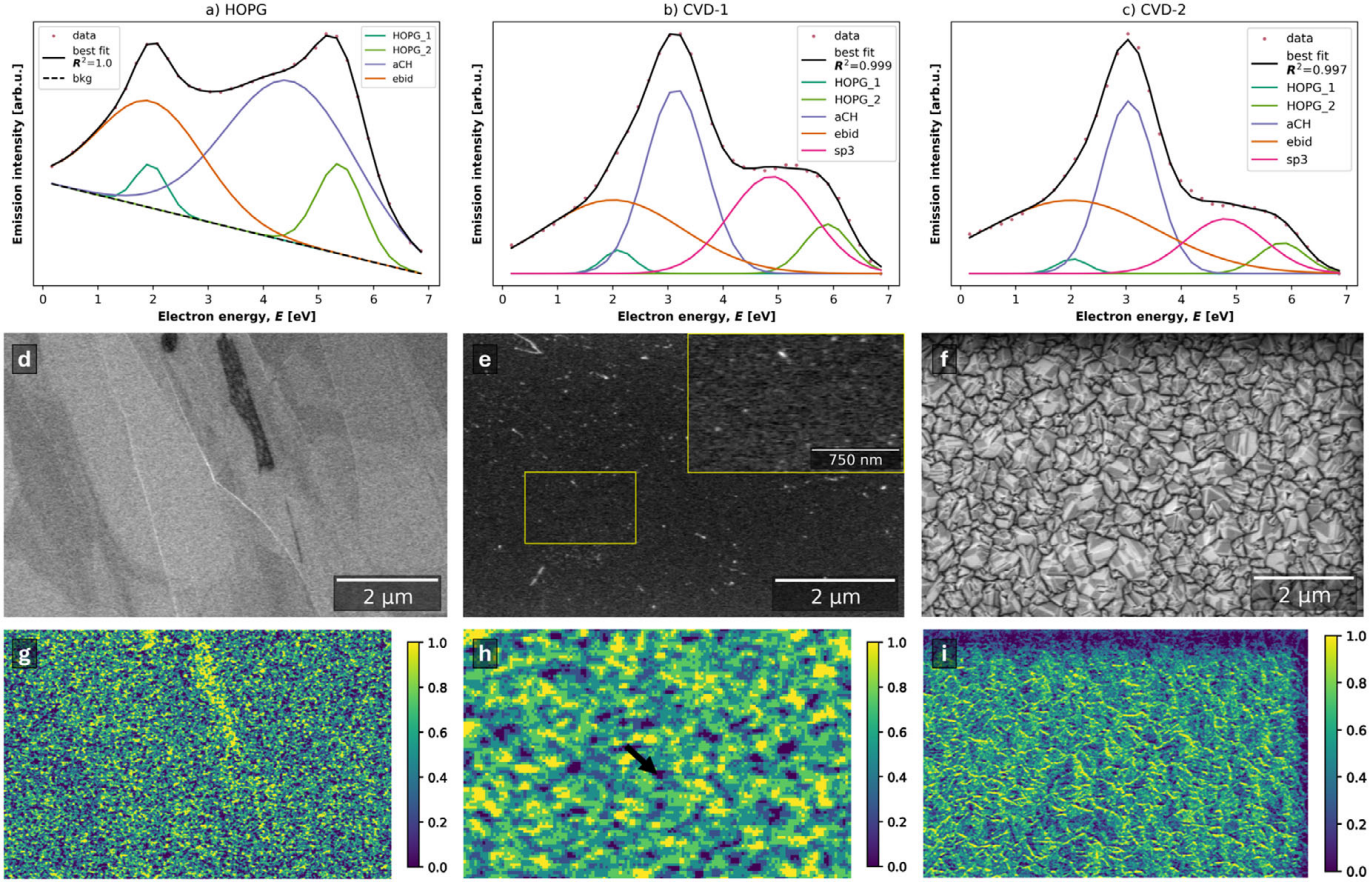
SE spectra of a) HOPG, b) CVD‐1, c) CVD‐2. SE‐spectrum of HOPG peak fit model with HOPG_1, HOPG_2, aCH, and ebid components (a), SE spectrum of HOPG peak fit model with additional sp^3^ component (b,c). SE images of HOPG, CVD‐1 and CVD‐2 respectively (d–f). Magnified region of interest from within the outline (e, inset). g) sp^2^ component maps, with ranges 5–6 eV for HOPG, h,i) 6–6.7 eV for CVD carbons.

The SE spectra of HOPG, CVD‐1 and CVD‐2 are shown in **Figure** **7**a–c. The Helios NanoLab 660/G (FEI) FIB‐SEM SEHI measurement parameters are listed in Table S1 (Supporting Information). The SEM chamber was plasma cleaned before SEHI measurements by five cycles of a 12 min plasma clean followed by 15 min pumping. After the cleaning cycles, the chamber was left under vacuum for >10 h before SEHI measurements. The procedure to produce average SE spectra is detailed in Section S2.3.1 (Supporting Information). Note the data in Figure 7 was produced from samples that were transferred from air involving a full chamber refill. CVD carbon spectra were measured with 12.50 and 25.00 pA beam currents to assess the influence of specimen charging on the resulting spectra. The 25.00 pA spectra had ±0.19 eV shifts in the first peak maximum, indicating minimal specimen charging with the 12.50 pA beam current condition (Figure S6, Supporting Information).

For fitting the SE spectrum of freshly exfoliated HOPG, initial values of peak centers of four components to the SE spectrum of HOPG were *HOPG_1*, *HOPG_2*, *aCH*, and *ebid*. Fitting model initial values and constraints are provided in Section S2.3.3 (Supporting Information). Four components were chosen based on: SE spectra of freshly exfoliated and surface treated HOPG (from SEHI),^[^ ^42^ ^]^ a series of SE spectra of carbon during graphitization,^[^ ^49^ ^]^ and a comparison of SE spectra from graphitic carbons.^[^ ^48^ ^]^ There is evidence of adventitious amorphous carbon contamination on HOPG surfaces from nano‐FTIR in Figure S10, Section S2.4 (Supporting Information), with absorbance at 1470 cm^−1^. Li et al. observed amorphous carbon contamination of graphene and graphite in air via Raman spectroscopy and XPS.^[^ ^55^ ^]^ The amorphous carbon contamination observed here could occur after the exfoliation of the HOPG specimen which takes place in air, and during the specimen loading period while the SEM chamber pumps to vacuum (imaging was carried out at a pressure of <0.30 mPa).

In the peak fit of the SE spectrum for HOPG (Figure 7a), the *HOPG_1* and *HOPG_2* peaks are centered at 1.96 and 5.39 eV respectively, with *aCH* at 4.50 eV and *ebid* at 1.95 eV.

The fitted *HOPG_1* and *aCH* component centers (shown in **Table** **4**.) are close to ranges reported previously for SEHI derived SE spectroscopy studies of carbon: Abrams et al. giving ranges 1.5–3.0 eV for sp^2^, 3.0–4.4 eV for *aCH*; 4.4–6.1 eV for sp^3^,^[^ ^42^ ^]^ and Farr et al. giving 2.9–4.3 eV for *aCH*.^[^ ^10^ ^]^ It should be noted however, that these studies only identified differences between SE spectra in the most intense emission regions, and the peak fitting analysis carried out here compared emission across the full energy range of the spectrum by producing a model with a measure of goodness of fit (*R* ^2^). The *HOPG_2* and *aCH* contributions are present in SE spectrum of freshly exfoliated HOPG in Abrams et al.,^[^ ^42^ ^]^ but are not evaluated by the maximum emission analysis.

**Table 4:** Peak centers from peak fits to SE spectra of reference materials. Average value ± standard deviation from repeats.

Material	Peak centres [eV]			
	HOPG_1	HOPG_2	*aCH*	sp^3^
HOPG	1.96 ± 0.01	5.39 ± 0.01	4.50 ± 0.00	–
CVD‐1	2.09 ± 0.15	5.89 ± 0.04	3.13 ± 0.12	4.87 ± 0.08
CVD‐2	2.03 ± 0.07	5.82 ± 0.02	3.05 ± 0.06	4.80 ± 0.00

Given there is a mix of sp^2^ and sp^3^ in both CVD‐1 and CVD‐2 carbon materials (as identified by XPS and Raman spectroscopy), an *sp* ^3^ component was added to the existing HOPG model for fitting SE spectra of these references. The *Amp*_HOPG_2_:*Amp*_HOPG_1_ ratio and sigma values for *HOPG_1* and *HOPG_2* components were fixed to equal values returned by the HOPG fit. Initial fit parameters and constraints are included in Section S2.3.3 (Supporting Information). The *HOPG*, *sp* ^3^, *aCH*, and *ebid* model delivered good fits to the average spectra of CVD‐1 and CVD‐2 carbon (Figure 7b,c). The *aCH* peak center shifted lower to 3.13 and 3.05 eV in fits of CVD‐1 and CVD‐2 carbon respectively, within ranges previously reported.^[^ ^10^, ^42^ ^]^

The SE spectra of HOPG, CVD‐1 and CVD‐2 carbon appeared less sensitive to differences in amorphous carbon of graphitic and tetrahedral character, with satisfactory fits obtained using just one component for *aCH* (as opposed to both *D* and *aC* contributions in Raman spectrum fits). Differences identified between SE spectra of CVD‐1 and CVD‐2 carbon relating to disordered and amorphous carbon were the *aCH* peak centers (Table 4) and FWHM of 1.27 ± 0.08 eV (CVD‐1) and 1.15 ± 0.02 eV (CVD‐2). The information depth for SEHI is closer to that of XPS than that of Raman. The discrepancy between Raman and XPS for the CVD‐2 film points towards an sp^2^ reach layer covering diamond that is present in the CVD‐2 film according to the Raman spectra. For comparing sp^2^ bonded carbon content between the reference materials, metrics for sp^2^% and sp^2^/sp^3^ were calculated. sp^2^% is *HOPG*_total_/*Area*_spec_ where *HOPG*_total_ is the sum of *HOPG_1* and *HOPG_2* peak areas and *Area*_spec_ is the area of all components excluding the *ebid* component. The sp^2^% values from SEHI differentiated between HOPG and CVD carbons as shown in **Table** **5**. However, there was no distinct difference in sp^2^% and sp^2^/sp^3^ ratio between CVD‐1 and CVD‐2 as measured by SEHI. At present we cannot exclude the possibility that defects to the sp^2^ bonding could be responsible for the change in the center position of the fitted peaks.

**Table 5:** Peak areas and peak area ratios from fits to SE spectra of reference materials. Average value ± standard deviation from repeats. The *ebid* component area is not included in the *Area*_spec_.

Material	Peak areas			Peak area ratios	
	HOPG_total_	aCH	sp^3^	$\frac{\mathrm{HOP}G_{\mathrm{total}}}{\mathrm{Are}a_{\mathrm{spec}}}$	$\frac{\mathrm{HOP}G_{\mathrm{total}}}{sp^{3}}$
HOPG	3.10 ± 0.18	11.18 ± 0.64	–	21.77 ± 1.78	–
CVD‐1	1.54 ± 0.14	5.56 ± 0.87	4.00 ± 0.66	13.89 ± 2.46	0.39 ± 0.11
CVD‐2	0.99 ± 0.10	4.64 ± 0.10	2.08 ± 0.04	12.86 ± 1.39	0.48 ± 0.05

Figure 7g–i is produced from emissions in the *HOPG_2* component range, chosen to indicate sp^2^ carbon distribution in the reference materials. The dark region on the HOPG surface in Figure 7d is therefore related to sp^2^ carbon where the 5‐6 eV emission range is brightest (Figure 7g). The distribution of sp^2^ in CVD‐1 (Figure 7h) appears to be in the form of nano‐domains distributed throughout the material, which matches the description of sp^2^ clusters in disordered carbons by Ferrari et al.^[^ ^35^ ^]^ CVD‐2 shows a localization of sp^2^ carbon to the boundaries between crystallite structures (Figure 7i). To further investigate the distribution of sp^3^ carbon and aCH in relation to sp^2^ in the CVD materials, aCH and sp^3^ channels were plotted in Figures S4 and S5 (Supporting Information) for CVD‐1 and CVD‐2 respectively. In CVD‐1, the aCH channel shows EBID around the edge of the field of view. There are also micron‐scale aCH contaminants, absent in the sp^2^ and sp^3^ channels (which show a homogeneous nano‐scale distribution of sp^2^ and sp^3^ domains). In CVD‐2, aCH is distributed along with sp^3^ in the crystallites, while sp^2^ is distributed between crystallites. None of the components show an increase in the contamination square region at the top of the field of view.

**Figure 8:**
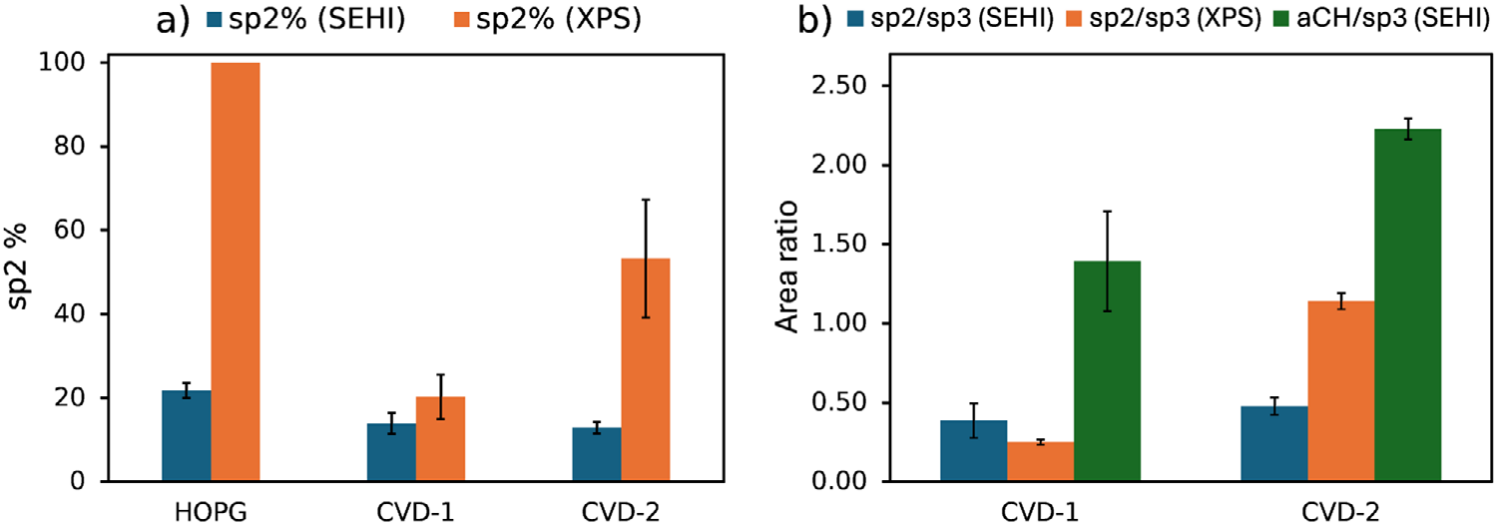
Comparison of fit metric outputs from SE spectra from SEHI and XPS spectra of reference materials. a) sp^2^%. b) sp^2^/sp^3^ (from SEHI and XPS) and aCH/sp^3^ (from SEHI).

The comparison between SEHI and XPS is warranted as the information depth is similar. The mean escape depth of SEs from amorphous carbon was calculated to be as little as 0.77 nm by Zou et al.,^[^ ^18^ ^]^ while Ono and Kanaya calculated mean escape depths of 11 and 4.8 nm for hydrogenated and graphitic carbon respectively.^[^ ^17^ ^]^ In the case of graphite therefore, the SE spectrum may be generated within the first ≈14 graphene layers versus ≈8 graphene layers for XPS. The approach to model the spectrum of HOPG and add components to model the disordered CVD carbons is also common between the fitting workflows. The sp^2^% and sp^2^/sp^3^ values are given in Table 5 and plotted versus comparable metrics from XPS in **Figure** **8**.

SEHI identified significant change in the sp^2^% between HOPG and CVD carbon reference materials. XPS identified the largest change in sp^2^% between the samples, giving significant changes in sp^2^ content between the CVD specimens, which SEHI did not. The lower sp^2^% in SEHI (Figure 8a) is expected as non‐sp^2^ components were fitted for EBID and aCH, whereas the XPS HOPG model is assumed to be 100% sp^2^. The sp^2^% metrics from fits of CVD materials spectra also deviate substantially from the values from XPS fitting. Given the inclusion of *aCH* in the graphitic carbon model, it is therefore expected that the sp^2^/sp^3^ proportion should be suppressed versus XPS measurements (as was the case for CVD‐2) and that *aCH* should be a high proportion in models of the disordered carbons (Figure 8b). In these comparisons, the SEHI metrics are calculated from a proportion of fitted component peak areas. An alternative approach could use the associated component map areas (plotted in Figures S4 and S5, Supporting Information for CVD‐1 and CVD‐2 respectively) to give component areal coverage %, as was the analysis approach in Section 5.2.2.

## 4 Influence of Experimental Parameters on Energy Filtered (EF)‐ Scanning Electron Microscope (SEM) Derived Secondary Electron (SE) Spectra

### 4.1 Influence of Instrument and Energy Calibration of EF‐SEM for SE Spectroscopy

To perform SEHI in different instruments, the energy axis must be calibrated in each instrument as differences in the hardware can influence the energy filtering performance. Differences in energy filtering performance can also arise from normal operation conditions. In plasma (P)FIB‐SEMs, material milled from the sample and may be re‐deposited elsewhere, including on/in column components.^[^ ^60^ ^]^

The low‐pass energy filter can be calibrated by stage biasing experiments. A detailed description of the stage biasing experiments can be found in refs. [13, 61]. Emitted electrons were accelerated by applying a negative stage bias voltage which was stepped between −5 and 0 V. The differences in energy shift between “S‐curves” were used to calculate an energy calibration coefficient.

The stage biasing experiment reported in ref. [13] was used to calibrate energy filtering of five FIB‐SEMs with the Elstar electron optics column (FEI/Thermo Fisher Scientific). Figure S7A–C (Supporting Information) shows the “S‐curve” responses and factor and shift calculations for three Ga^+^ FIB‐SEMs followed by a comparison to the reference SE spectrum from HOPG measured with FIB‐SEM A (Helios NanoLab 660/G). FIB‐SEM A, B, C have similar characteristics, with calibration factors of −0.40, −0.39, and −0.33 eV V^−1^, respectively. Calibration of Xe^+^ (P)FIB‐SEMs D and E was carried out by the stage biasing experiment as for A–C (Figure S8, Supporting Information). However, a method of energy calibration using only the 0 V stage bias response to CVD‐1 was used, as the PFIB‐SEMs S‐curve shift showed a non‐linear response to stage bias voltage steps. The calibration factor from A was used, plus a shift that would align the first peak in the CVD‐1 specimen. This peak is not affected by the high energy cutoff (onset may vary between (P)FIB‐SEMs) as the higher energy peak centers may be (Figure S9, Supporting Information).

**Figure 9:**
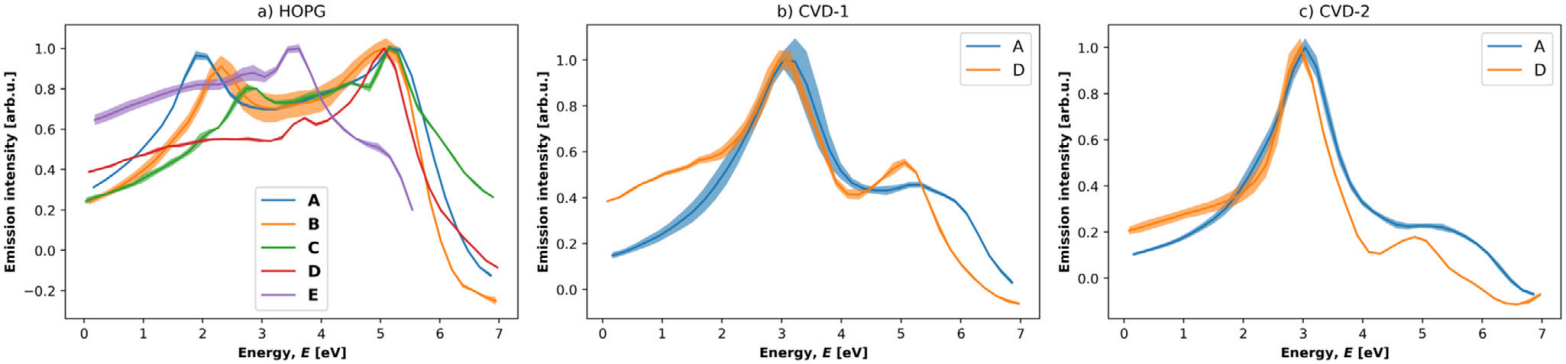
a) SE spectra of HOPG measured by (P)FIB‐SEMs with Elstar electron beam columns at five institutions, using energy calibration values as shown above. SE spectra of b) CVD‐1 and c) CVD‐2 carbon measured by (P)FIB‐SEMs A and D. Shaded regions represent average spectrum ± standard deviation. SE spectra area normalized to their maximum value.

SE spectra from HOPG for all systems trialed are plotted in **Figure** **9**a. SE spectra of CVD‐1 and CVD‐2 carbon as measured using FIB‐SEM A (Helios NanoLab 660/G) and PFIB‐SEM D (Helios G4 PFIB CXe) systems are plotted in Figure 9b.

The PFIB‐SEMs D and E have a feature centered at ≈3 eV, but much lower than the *aCH* peak center from HOPG fits. The second peak (previously reported at ≈5.5 eV) is visible in the HOPG SE spectrum from D. The high energy cut‐off above ≈6 eV has an earlier cutoff than FIB‐SEMs A, B, and C (hence why the second HOPG peak should not be used for energy shift calibration). The PFIB‐SEM E does not show first or second peak positions consistent with the results and models of HOPG spectra.

Nevertheless, the SE spectra of CVD‐1 and CVD‐2 carbon measured by the PFIB‐SEM D do show response to the difference in carbon character which is consistent with the SE spectra of the materials measured by FIB‐SEM A (Figure 9b,c). First peak centers are consistent, as well as first peak FWHM and intensity ratio change between the materials, albeit PFIB‐SEM D has a more abrupt high energy cutoff which changes the appearance of the second peak.

The findings indicate that energy filtering characteristics must be monitored as they can change through normal operation. PFIB (Xe^+^ beam) had a sputter rate ≈1.5× the FIB (Ga^+^ beam) when milling Si.^[^ ^62^ ^]^ Large volumes of material removed during specimen preparation may result in larger quantities of material that might be deposited elsewhere in the instrument, leading to subtle changes in electron optic performance especially in the low energy region. PFIB‐SEMs, which mill larger volumes, showed more variation in the low energy range energy filtering. The stage biasing experiment provides a means to compare and monitor energy filtering characteristics, to aid reproducibility and interpretation of SE spectra produced by SEHI. Further refinements of calibration methods in future could potentially lead to minimization of differences between instruments, such as using ancillary equipment to produce more accurate stage/specimen bias voltages.^[^ ^61^ ^]^ Further development of add on SE spectrometers, such as the Bessel‐box electron spectrometer design or other SEM add‐on spectrometers,^[^ ^53^, ^63^ ^]^ may also allow SEHI in the same area by two separate detectors to compare EF characteristics and to cross‐check calibration of the systems.

### 4.2 Minimizing the Effects of Electron Beam Induced Deposition (EBID) on SEHI Analysis

In this study, the extent of EBID contamination was monitored by SEHI in situ. First, we compared the SE spectrum contamination peak intensity to the height of contamination built up. Then we performed a sequential SEHI measurement to monitor the growth rate of contamination in various chamber conditions.

For SEHI measurements, the impact of EBID contamination was twofold: obscuration of surface features (which impacts conventional SE imaging), and modification of the surface chemistry. It is likely that the surface of the material under investigation will be modified by, and during, the SEHI measurement. The aim of this section is to understand the character of the EBID contamination, to determine its extent and evolution throughout the measurement, and to test approaches to reduce the extent of EBID contamination during SE imaging and SE spectroscopy.

The effect of EBID contamination was observed in electron microscopy particularly during surface sensitive and high‐resolution analyses like backscatter electron yield and imaging analyses or high‐resolution SE imaging.^[^ ^64^, ^65^ ^]^ Adventitious surface contamination and EBID contamination have therefore been considered throughout SE spectroscopy experimental design by monitoring EBID contamination during SEHI measurements in FIB‐SEM or surface pre‐preparation.^[^ ^52^, ^66^ ^]^

Studies related the growth of a surface layer of adventitious contamination to electron beam exposure, chamber vacuum level and specimen characteristics.^[^ ^42^, ^46^ ^]^ The mechanism by which surface contamination is deposited by electron beam interactions can be described as a stepwise process:

1. Molecules adsorb to and desorb from surfaces of the specimen and microscope chamber by van der Waals forces. The concentration of specimen surface adsorbed molecules is dependent on chamber pressure, specimen characteristics and beam induced desorption.
2. Irradiation and emission electrons ionize adsorbed molecules in the area of the specimen surface being scanned.
3. Bonds form between the surface and ionized contaminant molecules.
4. The region of contamination is now deficient in adsorbed molecules. Adsorbed molecules diffuse to the scanned area to maintain a concentration equilibrium.

The equation which incorporates steps 1–3 to describe the number of contaminant molecules, *n*_cont_, bonding to the surface per unit time and unit area was developed by Müller (with an additional electron energy, *E*_0_,^[^ ^67^ ^]^ dependent term for contaminant cracking and crosslinking, *σ*_c_, and desorption, *σ*_d_),^[^ ^46^ ^]^

(1) $$n_{\mathrm{cont}}=\frac{p}{\sqrt{2\pi mk_{B}T}}\cdot \left(\sigma_{c}-\sigma_{d}\right)\left(E_{0}\right)\cdot n_{e}$$

where the first factor represents the density of adsorbed molecules per unit time and unit area where *p* is partial pressure, *m* is molecular mass, *k*_B_ is the Boltzmann constant and *T* is temperature. The second factor is the electron energy *E*_0_ dependent cross‐sections of contaminant cracking and crosslinking per unit time and unit area, *σ*_c_, minus the cross‐section of contaminant desorption, *σ*_d_, per unit time and unit area. The final factor *n*_e_ is the number of electrons per unit time and unit area.

As a result, the extent of contamination in SEM over an image area and acquisition time is dependent on chamber temperature, partial pressure, primary electron energy, and electron dose; the latter three of which can be measured reliably in SEM image acquisition.

In SEM, the electron dose [cm^−2^] received during image acquisition, *D*_img_, is given by

(2) $$D_{\mathrm{img}}=\frac{I_{0}\cdot t_{\mathrm{dwell}}\cdot n_{\mathrm{px}}\cdot \left(n_{\mathrm{average}}+n_{\mathrm{integrate}}\right)}{A}$$

where beam and imaging parameters are: *I*_0_ the beam current [A], *t*_dwell_ the pixel dwell time [s], *n*_px_ the number of pixels in the image, (*n*_average_+*n*_intergrate_) the number of frames averaged and/or integrated per image, and *A* the area of the image [m^−2^].^[^ ^68^ ^]^

The electron dose received during SEHI data volume acquisition, *D*_spec_, is then a factor of the number of image slices in the hyperspectral image volume, *n*_img_.

(3) $$D_{\mathrm{spec}}=D_{\mathrm{img}}\cdot n_{\mathrm{img}}$$

Assuming a constant temperature and a primary electron energy of 1 keV, we compare the electron dose received by the specimen in the image area to the extent of contamination at a range of chamber pressures. Primary electron bombardment does increase the temperature of the specimen local to the beam, however in SEHI conditions (see Table S1, Supporting Information), the effect is believed to be negligible based on comparison to conditions simulated by Zhang and Zhang.^[^ ^69^ ^]^ We also assume that factors related to the molecular mass of contaminant species, *m*, and cross‐sections of cracking and crosslinking and desorption (*σ*_c_ and *σ*_d_) are modified by chamber plasma cleaning. The relationship between *n*_cont_ and *n*_e_ (i.e., *D*_spec_) is linear when *p*, *m*, *T*, and *E*_0_ are constant (Equation (1)).

Experiments that investigate the rate of EBID contamination must have a means to quantify the extent of EBID contamination produced. Hugenschmidt et al. measured the thickness of contamination by in situ transmission mode electron microscopy and ex situ atomic force microscopy (AFM),^[^ ^46^ ^]^ while Li and Joy measured the volume of contamination by in situ SE imaging.^[^ ^70^ ^]^ Hirsch et al. used in situ backscatter electron image contrast as a proxy for contamination thickness.^[^ ^71^ ^]^ In LV‐SEM conditions, studies have monitored the extent of contamination versus electron dose by measuring the height of the contamination produced with ex situ AFM.^[^ ^9^, ^42^ ^]^

**Figure 10:**
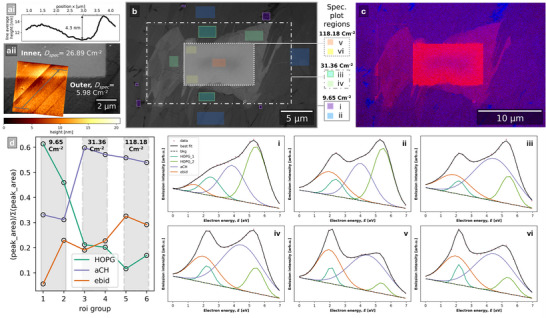
a‐i) Line profile of height parallel with grain along line in a‐ii) overlaid AFM height map, with width of end caps. a‐ii) SE image of two electron dose regions: inner *D*_spec_ = 26.89 cm^−2^, outer *D*_spec_ = 5.98 cm^−2^, with overlaid AFM height map region of 5 µm × 5 µm that intersects both electron dose conditions. The height difference step between outer and inner dose regions is +4.3 nm. b) SE image of the HOPG surface where the central region ≈10 µm wide (bounded by dotted line) received an electron dose of 118.18 cm^−2^, the outer central region (bounded by the dashed‐dotted line) received an electron dose of 31.36 cm^−2^, and the outside region ≈40 µm wide (bounded by the solid line) received a 9.65 cm^−2^ electron dose. Color overlays are grouped regions (i–vi) from which spectra are plotted from. Regions were selected by hand to represent a range of EBID conditions in the field of view. c) Color secondary electron hyperspectral imaging (CSEHI) image where energy ranges 1.2–3.2 and 4.2–6.2 eV are assigned to red and blue color channels respectively. d) Plot of peak area ratios for the total of HOPG peaks, aCH, and ebid peak. The shaded bands show electron dose conditions which regions received. i–vi) SE spectra from regions in (b) with HOPG fitting model of HOPG_1, HOPG_2, aCH, and ebid components.

First, we produced an AFM height map of an area that intersected two electron dose conditions. There was a +4.3 nm step between the outer region which received a lower electron dose of 5.98 cm^−2^ to the inner region which received an electron dose of 26.89 cm^−2^ (**Figure** **10**a).

The extent of EBID is expected to be inhomogeneous depending on the scan area geometry. Hugenschmidt et al. observed a thicker contamination at the edges of irradiated areas, which was explained by the diffusion of adsorbed molecules across the surface from the periphery after depletion of adsorbed contaminants in the irradiated region.^[^ ^46^, ^72^ ^]^

EBID contamination thickness is also modified by scanning patterns (as in SEM). Raster scanning allows time for replenishment of adsorbed molecules from the edges of the scan area to the center. Shorter pixel dwell times and interlaced scanning results in less time for replenishment of contaminants to the center of the scanned region by diffusion. Variation around the perimeter of the scan is also common, and results from dwell at the top and left edges of the frame between frame and line scans. O'Connell observed these variations in contamination height around the perimeter of the scan region by AFM height mapping a 5 µm × 5 µm square area scanned by a 1 keV primary energy beam.^[^ ^73^ ^]^

Scan effects were visible in a second inner–outer electron dose condition experiment. At low electron doses, i.e., outside the central ≈10 µm wide region (Figure 10b), the rate of EBID was dependent on features of the HOPG surface. In Figure 10b, the CSEHI image was colored by assigning emissions in energy ranges to color channels in an RGB image (software available).^[^ ^74^ ^]^ Energy ranges are 1.2–3.2 eV (red) and 4.2–6.2 eV (blue) which correspond to *ebid* and *HOPG_2* peak centers ± 1 eV in the whole field of view SE spectrum. Regions of interest were selected to represent various EBID conditions in the field of view from which SE spectra were plotted (Figure 10i–vi). The SE spectra show an evolution of spectrum shape and fits with decreasing *HOPG*_total_ peak area as a proportion of the total SE spectrum area in three dose condition regions. The fitted *ebid* peak area was at a minimum (0.74) in region (i) spectrum, and reached a maximum in region (vi) (inside corners of the central contamination square, Figure 10b).

The variation of EBID contamination in the low to mid electron dose regions (9.65 and 31.36 cm^−2^) maps to underlying grains of the HOPG. The observed variation in EBID contamination could result from grain dependent cross‐sections for contaminant cracking, crosslinking and desorption, and/or diffusion rate of contaminants into the depleted deposition region. Lower *ebid* component contribution to the region (i) indicates that the beam energy (1 keV) and electron dose condition results in lower cracking and crosslinking cross‐sections.

Meanwhile in the central region, the underlying HOPG grain morphology is not visible due to a greater extent of EBID contamination. The highest proportion of *ebid* component area occurs in region (vi) at the inner corners of the central scan region, where contaminants are replenished by surface diffusion. There is less contamination in the central region (v) where contaminants are replenished solely by adsorption from the chamber gas.

In the SE spectrum of HOPG, the increase in first peak intensity at ≈2 eV with electron dose is therefore related to EBID contamination. The decrease in the second peak emission intensity at ≈5.5 eV is related to loss of HOPG SE spectrum signal by an overlayer of contamination. Using the first and second peak intensities as a measure of the extent of contamination, the evolution of contamination versus electron dose in various chamber conditions was assessed by sequentially collecting SEHI data volumes in the same area. The experiment detailed in Figure S11 (Supporting Information), results of which are presented in parts 3.2.1 and 3.2.2, averages the SE spectrum over the whole field of view to yield a spatially averaged measure of EBID contamination.

The following section presents results of the sequential SEHI measurement experiment which tracks the emission peak intensity evolution in SE spectra of HOPG. The experiment assesses how conditions such as chamber pressure and cleaning vary the growth rate of contamination.

#### 4.2.1 Effect of Chamber Pressure on Contamination

Since the rate of adsorption of contaminants to the specimen surface is related to the chamber pressure (Equation (1)) the reduction of chamber pressure should result in more reproducible SE spectra with lower levels of EBID contamination on the specimen surface.

##### Chamber Airlock

Specimen transfer preceded the sequential SEHI measurements. In one case, the HOPG specimen was transferred into the SEM chamber by filling the chamber with nitrogen gas, opening the chamber to insert the specimen; then evacuating the chamber. In the second, the specimen was transferred via an intermediate airlock chamber. The airlock fitted chamber maintained an average vacuum level of 0.08 mPa during measurements versus the conventional chamber at 0.30 mPa.

**Figure 11:**
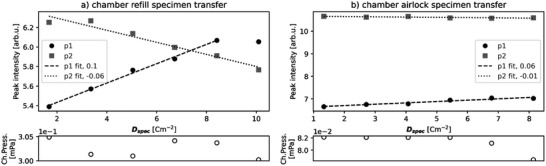
Plots of peak intensity versus *D*_spec_ [cm^−2^] with chamber pressure (Ch.Press.) [mPa] after two methods of specimen transfer. a) Chamber refill with nitrogen gas and chamber door opening to ambient conditions. b) Airlock. Average chamber pressure during the measurement series was 0.30 mPa (a) and 0.08 mPa (b).

SE spectra of HOPG produced by the experiment detailed in Section S2.5 (Supporting Information) were analyzed as follows: the peak intensity of the two peaks in the SE spectrum of HOPG were plotted versus cumulative electron dose per SE spectrum, *D*_spec_. As electron dose increased, the first peak intensity increased and the second peak intensity decreased (p1 and p2 in **Figure** **11**). First peak intensity is shown to increase with EBID contamination as shown in Figure 10, while the loss of second peak intensity is associated with contamination covering the HOPG surface.

Given that variables: temperature and beam energy were kept constant while electron dose was increased, and chamber pressure within a small range, the relation between contaminant deposition and electron dose is expected to be linear. Therefore, peak intensity versus *D*_spec_ was fitted as linear—with positive gradient for the EBID contamination associated first peak and negative for the HOPG surface associated second peak. The linear relationship may be broken (≈10 cm^−2^) as contaminants are depleted and the rate of replenishment of the scanned area contaminants by adsorption and diffusion does not match that of deposition. The shallower gradient in the airlock specimen transfer case indicates more reliable conditions for data collection.

##### Cryo‐SEM Anti‐Contaminator

**Figure 12:**
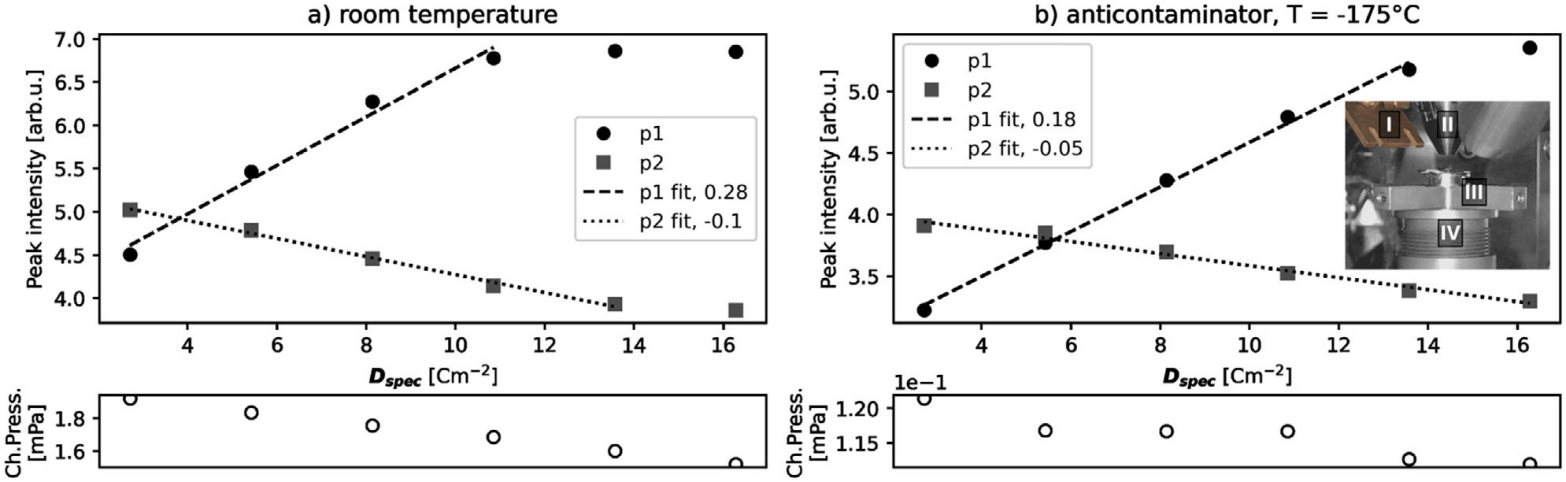
Plots of peak intensity versus *D*_spec_ [cm^−2^] with chamber pressure (Ch.Press.) [mPa] a) with anti‐contaminator at room temperature and b) with anti‐contaminator cooled to −175 °C. Inset: I, metal anti‐contaminator; II, pole piece; III, HOPG specimen on stub; IV, conventional specimen stage. Average chamber pressure during the measurement series was 1.72 mPa (a) and 0.12 mPa (b).

Here the “anti‐contaminator” from an in‐chamber cryo‐SEM apparatus (Quorum Technologies Ltd., UK) was at −175 °C during SEHI measurements, which had the effect of reducing chamber pressure. The anti‐contaminator is a metal plate which is cooled by the circulation of nitrogen gas. The anti‐contaminator is deployed parallel to the pole piece in the SEM chamber (visible in **Figure** **12b**, inset). When not in use, the anti‐contaminator folds away from the pole piece. Specimen cooling was not applied here, owing to arduous sample and instrument preparation and reduced stage movement **Figure** 12.

**Figure 13:**
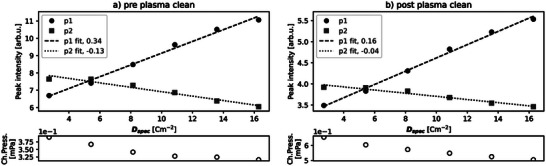
Plots of peak intensity versus *D*_spec_ [cm^−2^] with chamber pressure (Ch.Press.) [mPa] from a) pre‐plasma cleaning condition and b) post‐plasma cleaning of five sets of 15 min plasma followed by pumping to high vacuum for 10 min. Average chamber pressure during the measurement series was 0.34 mPa (a) and 0.57 mPa (b).

The anti‐contaminator is at a lower temperature than the specimen so that there is a concentration gradient that results in preferential adsorption of molecules on the anti‐contaminator compared to the specimen surface. Over time, the adsorption of gaseous molecules on the anti‐contaminator reduces the chamber vacuum level and therefore the concentration of molecules adsorbed to the specimen surface reduces.

Without cooling the cryo‐SEM anti‐contaminator, the chamber pressure during SEHI measurements was 1.50–1.90 mPa and with cryo‐SEM anti‐contaminator cooled to −175 °C, the chamber pressure was 0.11–0.12 mPa (see chamber pressure, ChPress., in plots Figure 12a,b).

SEHI data volumes were measured in the same area of a freshly exfoliated HOPG surface in two conditions, a) without cooling of the anti‐contaminator and b) with cooling to −175 °C of the anti‐contaminator. The anti‐contaminator condition shows a reduction in average chamber pressure during acquisition from 1.80 mPa (room temperature conditions) to 0.12 mPa (with anti‐contaminator). Sequential SEHI measurements in the same area cumulates electron dose in the measurement area.

In both conditions the evolution of peak intensities shows an initial linear relationship with *D*_spec_ until there is a plateau in first peak intensity. The cause of a plateau in the contamination peak intensity may be: i) the EBID contamination grows to a thickness that includes the whole SE escape depth; ii) the growth of EBID contamination lowers the local contaminant replenishment rate; iii) the change in surface condition changes the cross‐sections of adsorption, desorption, cracking and crosslinking.

The linear relationship is broken above ≈11 cm^−2^ in the room temperature condition (Figure 12a), versus ≈13.5 cm^−2^ in the anti‐contaminator condition (Figure 12b). Since contaminant replenishment is likely higher in the room temperature condition, this suggests that the EBID contamination has thickened to include the entire SE escape depth. The decrease in second peak intensity is associated with the reduction in escape depth that intersects with the underlying uncontaminated surface.

#### 4.2.2 Effect of Chamber Plasma Cleaning on Contamination

“Pre‐plasma clean” (**Figure** **13**) measurements were taken before a chamber plasma cleaning program as follows: air plasma was injected into the vacuum chamber for 15 min followed by pumping to high vacuum for 10 min. The combination of plasma cleaning and pumping was repeated five times. The chamber was left to pump to high vacuum overnight. Following the plasma cleaning, measurements in the “post‐plasma” condition were taken.

Chamber plasma cleaning reduced the first peak intensity gradient compared to pre‐plasma cleaning. The partial pressure in the post‐plasma clean condition is higher, yet the amount of EBID contamination was measured by SEHI to be lower.

Given beam energy, *E*_0_, and *D*_spec_ were consistent between the two conditions, we infer the makeup of molecules in the SEM chamber has changed between pre and post clean conditions. This effect has been observed in instruments with in‐vacuum‐line mass spectrometers which measured molecular mass pre and post chamber plasma cleans.^[^ ^75^ ^]^ According to Equation (1), a reduction in molecular mass increases the concentration of adsorbed contaminants, increasing the potential for EBID contamination. However, a change to the cross sections of adsorption, desorption, and cracking and crosslinking could counteract this effect.

### 4.3 Best Practices for SEHI Derived SE Spectroscopy

Following Section 4 of the article, the prerequisites for implementing SEHI and SEM‐derived SE‐spectroscopy are complete. An energy calibration was applied to the energy filtering in the Elstar column through a stage biasing experiment. FIB‐SEMs had similar energy filtering characteristics and resulting HOPG SE spectrum shape. The PFIB‐SEM systems in the test had different energy filtering characteristics, without linear response to stage bias steps in the experiment in Section 4.1. This could result from subtle changes to electron performance due to the increased amount of ion‐beam milled material deposition by PFIBs compared to FIBs, as well as the material types being milled. Therefore, it is recommended to regularly check the system's energy calibration, either by stage biasing experiment or measuring the SE spectrum of HOPG.

Following energy calibration, we assessed the extent of EBID contamination during SEHI measurements. SEHI measured EBID contamination in situ as the electron dose increased.

As predicted by models of EBID contamination growth, the rate of contaminant deposition varied through the irradiated area, with thicker contamination at sample edges due to contaminant replenishment by diffusion, and less contamination in the center of irradiated areas where the chamber residual gas is the main contributor of contaminants.^[^ ^46^, ^75^ ^]^ EBID contamination occurred at different rates on different grains of HOPG, indicating factors local to the specimen surface modified the rate of EBID. This result identified the EBID contamination component in SE spectra of HOPG at ≈2 eV.

The sequential SEHI measurement experiment was performed in various chamber conditions to compare the effect of pressure, load‐lock transfer, cryo‐SEM anti‐contaminator, and plasma cleaning. Chamber plasma cleaning influences the molecular species present in the chamber, probably reducing the molecular weight.^[^ ^75^ ^]^ Despite a higher chamber pressure in the post‐plasma condition (0.57 mPa compared to 0.34 mPa) the first peak fit gradient in the plasma cleaning case was 0.16 as opposed to 0.34 in the pre‐plasma cleaning condition. Since decreasing molecular weight, *m* (Equation (1)), would increase the number of adsorbed molecules, we conclude that in plasma‐cleaned conditions, the cross‐sections of (adsorbed) contaminant cracking and crosslinking are reduced. Furthermore, the lack of EBID in some areas of the HOPG surface in Figure 10a,b indicates that cracking and crosslinking cross‐section thresholds may not be exceeded due to combined surface, beam and contaminant conditions.

**Figure 14:**
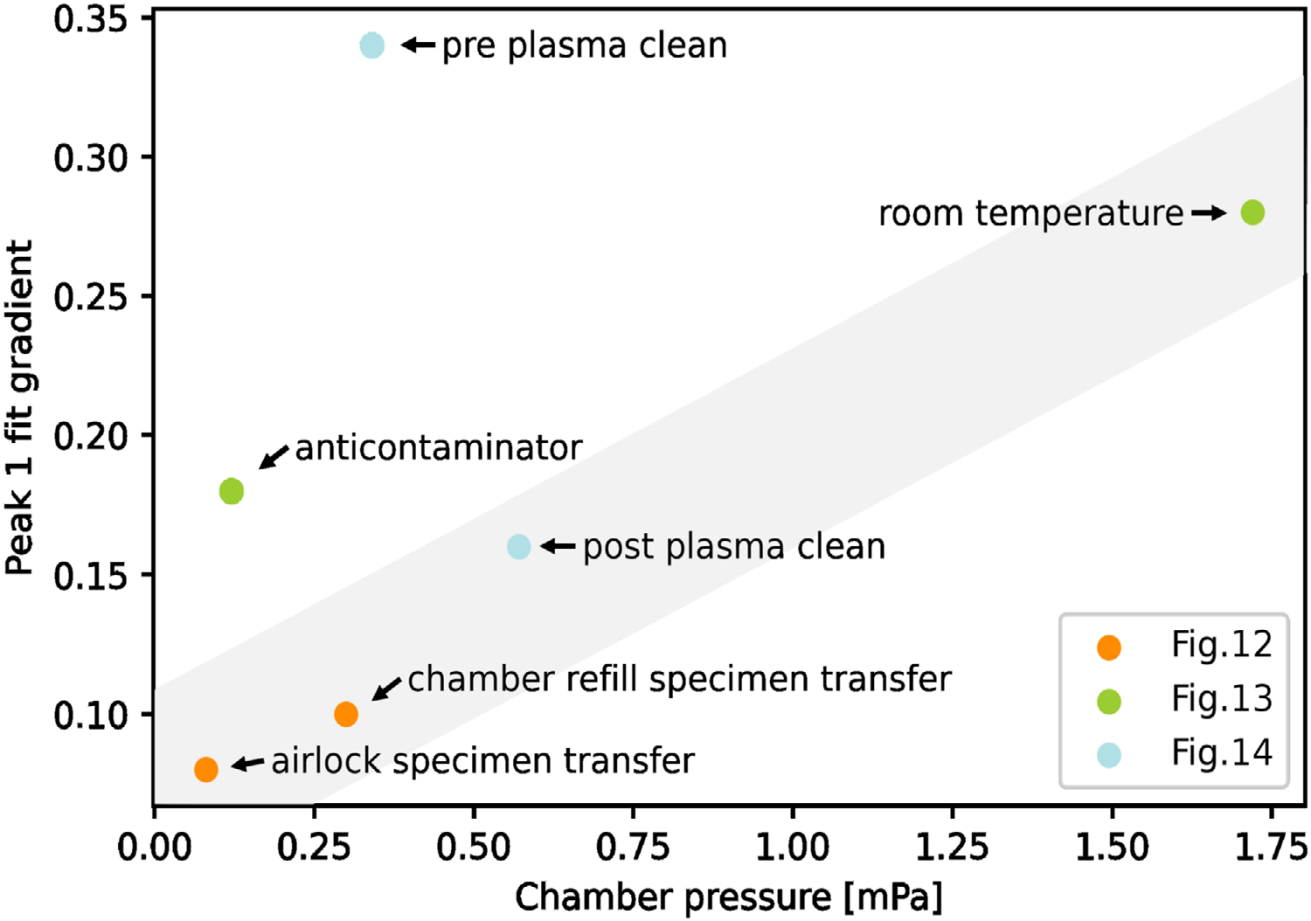
Summary plot of EBID contamination associated peak 1 versus *D*_spec_ fit gradient and chamber pressure from results presented in Figure 11: specimen loading conditions, Figure 12: anti‐contaminator versus room temperature, and Figure 13: pre‐ and post‐plasma cleaning of the SEM chamber. Shaded region represents “clean” chamber condition.

To conclude, the methods to reduce EBID contamination can be used in combination, but should start with chamber plasma cleaning, as gains in slowing EBID from plasma cleaning can outweigh those from reducing the vacuum level (see conditions within the “clean” chamber shaded region in **Figure** **14**). Once the makeup of contaminants has been modified by plasma cleaning, measures should be taken to reduce the vacuum level where possible, by longer evacuation times or, as was most effective here, by specimen transfer through an airlock chamber.

## 5 Application Materials

### 5.1 Carbon Coated Lithium Iron Phosphate

#### 5.1.1 Material Description and Significance

Production of LIB active materials has scaled to meet demand in electric vehicles and lesser energy storage applications. Lithium iron phosphate (LiFePO_4_, LFP) has a growing share of total LIB cathode material production, with wide ranging cost, material and energy impacts.^[^ ^76^ ^]^ Key to the material performance is the quality of a conductive carbon coating on primary particles, given LFP is an electronically insulating material with bulk electrical conductivity in the range of 10^−10^–10^−9^ S cm^−1^.^[^ ^77^ ^]^ The application of the carbon coating can improve bulk conductivity of powders to 10^−4^–10^−2^ S cm^−1^.^[^ ^78^ ^]^

In battery applications, the increase in LFP electrical conductivity correlates with an increase in specific capacity and rate performance.^[^ ^79^ ^]^ The requirements for performance in electric vehicles include not only sufficient specific energy but also stable performance at high rates of charge and discharge. LFP balances mid‐table specific energy with a comparatively low materials cost to give a competitive cell‐level cost of 150 $ kWh^−1^.^[^ ^77^ ^]^

The variation in conductivity post carbon coating indicates differences in the nature of the carbon within the coating. Graphitic carbon is the desired outcome owing to its high electronic conductivity, but the coating produced may contain amorphous carbons. Raman spectroscopy is commonly used to characterize the ordered (graphitic) to disordered (non‐graphitic) carbon ratio, averaged over the analysis area.^[^ ^2^, ^80^ ^]^

Given the thickness of optimized graphitic carbon coatings is on the nanoscale, HR‐TEM is often used to obtain information about the carbon coating character at the edges of particles and may indicate the thickness and quality of the coating by morphology analysis.^[^ ^2^, ^81^ ^]^ The HR‐TEM method has limitations in characterizing the coating, being a visual assay without mapping. The HFW of HR‐TEM images typically show a region of coating in a <100 nm HFW making it time consuming to achieve a representative assay of the material.^[^ ^81^ ^]^

#### 5.1.2 Characterization of Carbon Coating

A commercial carbon coated LFP material was characterized by SEHI and Raman spectroscopy.

**Figure 15:**
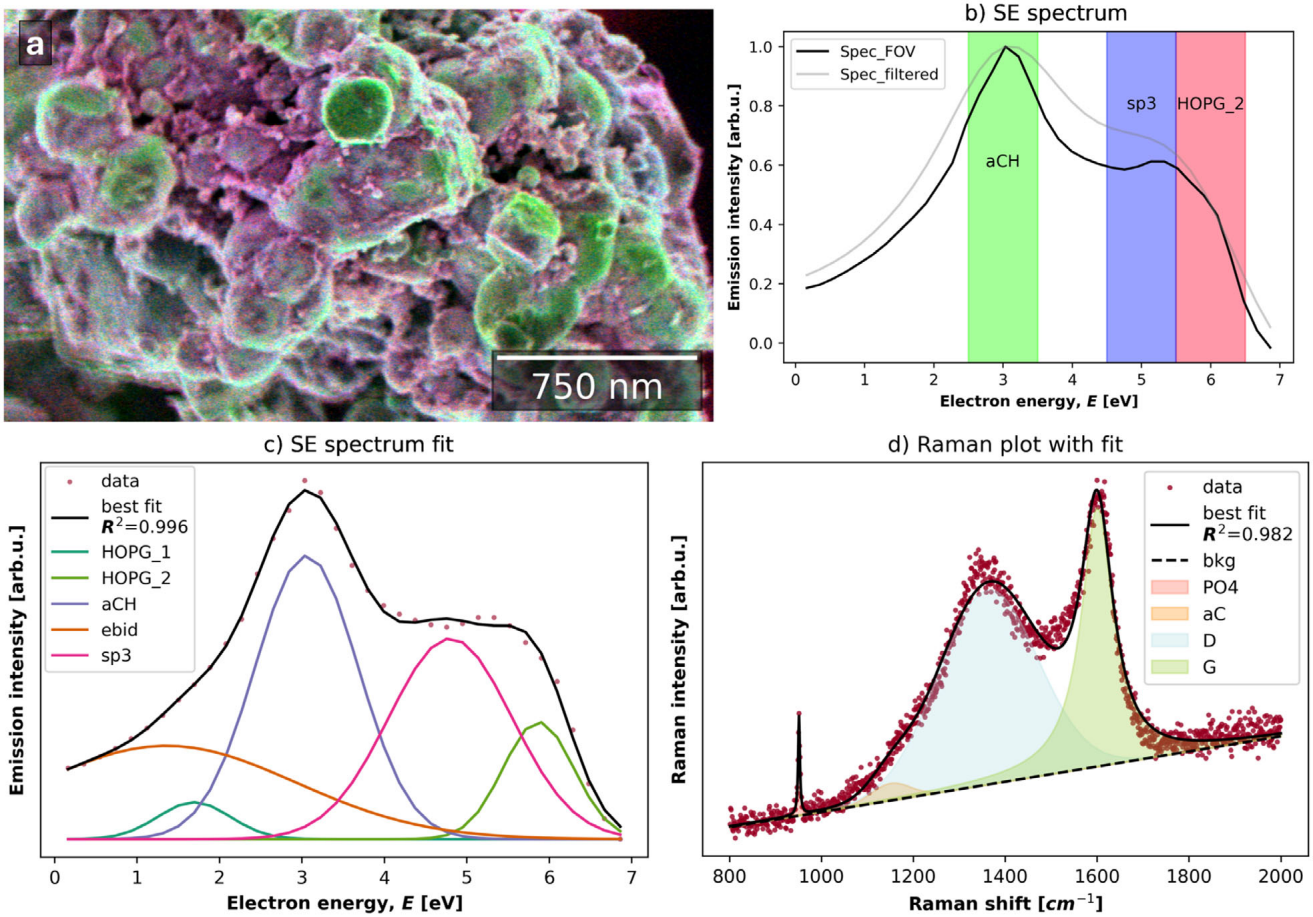
a) Color SEHI image of C‐LFP primary particles with disordered graphitic carbon coating visible in purple and amorphous carbon coating in green. b) SE spectrum and SE spectrum post 3D Gaussian filter with underlaid energy ranges assigned to the RGB channels of the image in (a), with corresponding component labels. c) SE spectrum fit using disordered graphitic carbon model. d) Raman spectrum of commercial LFP material with P─O bond vibration peak at ≈950 cm^−1^ fitted with PO4, aC, D, and G components.

The CSEHI image in **Figure** **15**a was produced by assigning energy ranges 5.5–6.5, 2.5–3.5, 4.5–5.5 eV to RGB image channels. Angular correction by image acquisition at two stage rotations was used (detailed in Section S3.1, Supporting Information). The *HOPG* plus *sp* ^3^ peak fitting model (developed in Section S3.2, Supporting Information) was used to fit the SE spectrum of the carbon coated LFP material (Figure 15c). Energy ranges (underlaid in the SE spectrum plot in Figure 15b correspond to *HOPG_2* (R), *aCH* (G) and *sp* ^3^ (B) components.

The dominant colors in the SEHI color image are green and purple (a mix of red and blue). A CSEHI image produced by an alternative color assignment order is provided in Figure S14 (Supporting Information) to aid readers with difficulty distinguishing red‐green color contrast. The green range indicates amorphous carbon while the red and blue ranges include emissions associated with disordered graphitic carbon.

The color image shows a network of disordered graphitic carbon coating on primary particles of LFP, with amorphous carbon “islands” between disordered graphitic regions. The amorphous carbon is dissociated from the disordered graphitic regions. The disordered graphitic coating network is also seen to have smooth and particulate morphologies. Interestingly, the ability to visualize regions of graphitic and amorphous carbon in this way could be useful in assessing how varying regions of graphitic conducting carbon versus amorphous carbon impacts on the resulting electrochemical properties.

The SE spectrum fitting yields *aCH/HOPG*_total_ and *sp3/HOPG*_total_ ratios of 2.69 and 2.27 respectively (where *HOPG*_total_ is the sum of *HOPG_1* and *HOPG_2* peak areas). This indicates a lower proportion of amorphous carbon and similar sp^3^ content compared to the CVD‐1 and CVD‐2 reference materials.

The nano‐thickness of carbon coating on the LFP material provides an opportunity to compare SE spectra with resonant Raman spectra of carbons from the similar surface depths. The Raman spectrum fit of LFP (Figure 15d) included a peak at ≈950 cm^−1^ associated with LFP P─O bond vibrations,^[^ ^82^ ^]^ and carbon *aC*, *D*, and *G* peaks indicative of a disordered graphitic carbon. The *A*_D_:*A*_G_ ratio is 1.55. The FWHM of the *D* and *G* peaks are 256.1 and 100.4 respectively. The *D*:*G* ratio indicates more disorder coordinated with carbon rings compared to CVD‐1 and CVD‐2 carbon while the FWHM of the *G* peak is lower, indicating larger graphitic regions.

CSEHI characterization gave a new perspective on carbon coating of LFP material, showing a graphitic carbon network amongst an amorphous carbon coating on LFP. The result from Raman spectroscopy spectrum fitting supports the SE spectrum fitting, by indicating graphitic domains separated by regions of disorder. Standard analysis of graphitic carbon coatings on LFP used HR‐TEM to image atomic planes of carbon at particle edges and therefore could not extend to the scale of primary particles and secondary particle assemblies.^[^ ^81^ ^]^ The image resolution measured by edge response is 5.4 nm (Figure S15, Supporting Information). The CSEHI image enabled the coating to be viewed at the scale of primary particles ≈300 nm in diameter, within a secondary particle assembly ≈2.4 µm in diameter.

### 5.2 Lithium‐Ion Battery Graphite Electrode

#### 5.2.1 Material Description and Significance

The graphite electrode is used as an anode in LIBs. The electrode (Cambridge Energy Solutions Ltd.) is a composite of graphite, CB conductive additive and a polymer binder blend of carboxymethyl cellulose (CMC) and styrene butadiene (SBR) rubber in a 93.2:2.5:2.5:1.8 ratio of masses. The electrode active material, CB and polymer binder were produced by slurry casting onto a copper current collector. Each carbon component serves a unique and specialized function. Graphite particles serve as Li‐ion intercalation material with ≈320 mAh g^−1^ specific capacity, the CMC and SBR polymer blend binder is compatible with aqueous solution slurry casting and has suitable toughness and deformation properties. All carbon components must be compatible with organic Li‐ion electrolytes.^[^ ^83^ ^]^

The ability to segment and map the graphite active material and carbon binder material in the LIB anode is valuable to battery materials development as it becomes possible to make a visual assay of the binder material distribution, which may be important in the development of binder polymers^[^ ^84^ ^]^ or novel electrode film manufacturing routes.^[^ ^85^ ^]^

#### 5.2.2 Characterization Workflow Guide for Color to Disordered Materials Identification

The following SEHI characterization serves as an example of how SEHI can be used as a tool to map surface chemical heterogeneity, and then identify chemical species.

**Figure 16:**
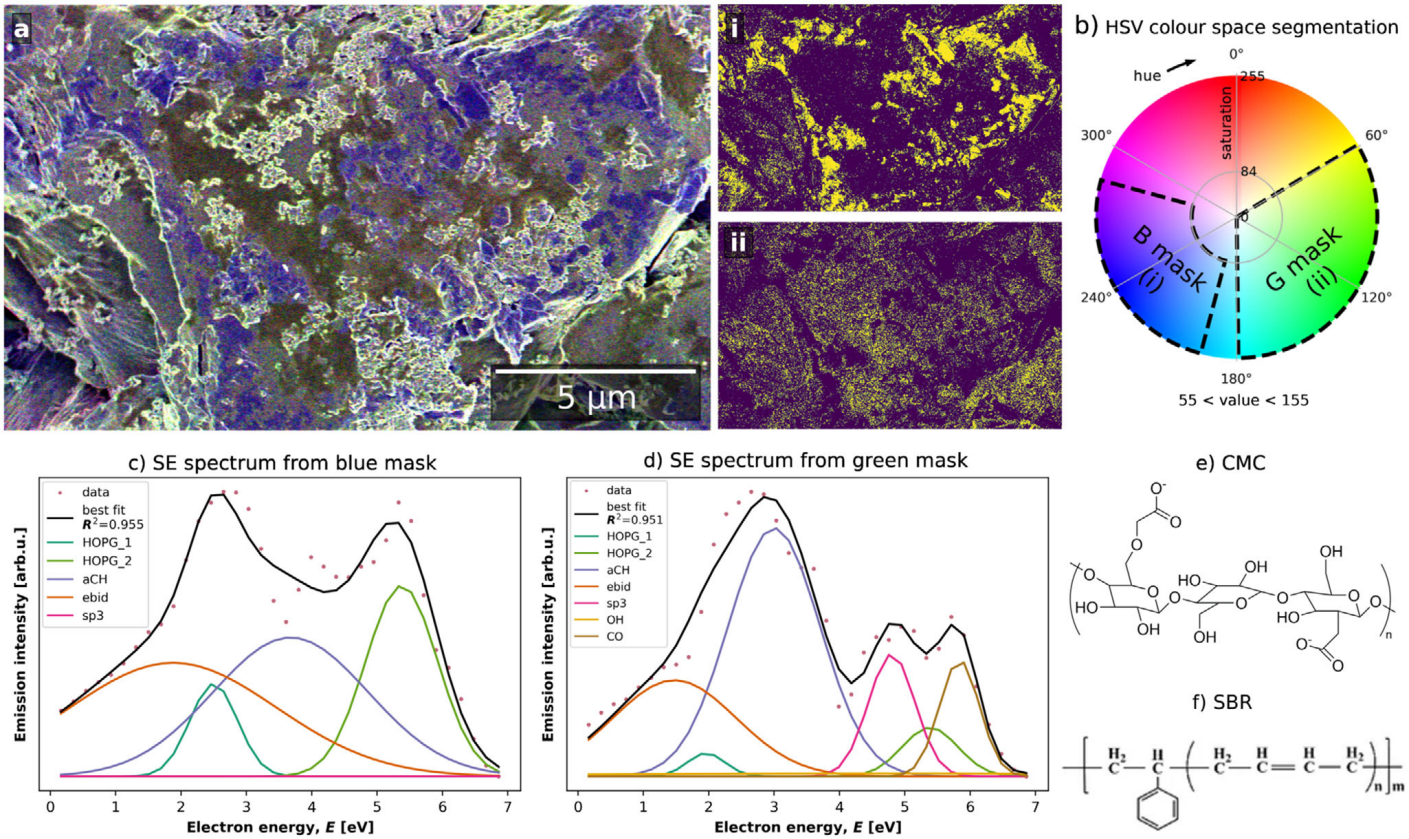
a) CSEHI image of graphite anode material from LIB. Masks of areas in (a) produced by selecting pixels with i) blue hue values, ii) green hue values. b) Hue; saturation; value (HSV) color space segmentation schematic. SE spectra from mask regions with fits using c) disordered graphite model and d) oxidized carbons model. e) CMC monomer. f) SBR monomer from Wang et al., Nanoscale Res. Lett. (2017). Reproduced under the terms of the CC BY 4.0 license.^[^ ^83^ ^]^

To quickly visualize heterogeneity, emissions in 0–2, 2–4, 4–6 eV energy ranges were assigned to red; green; blue (RGB) image ranges (**Figure** **16**a). Including a wide range of emissions in the image improved noise characteristics of the color image.

Thresholding the CSEHI image HSV color space (Figure 16b) produced the blue and green region masks in Figure 16i,ii. Spectra were produced from each segmented region and disordered graphitic and oxidized carbon fitting models were used to compare the graphitic, amorphous carbon and oxygen functional group character of the regions. The resulting SE spectrum from the blue region (Figure 16c) shows characteristics of the SE spectrum of graphite and is fitted with the disordered graphitic carbon model developed in Section 3.2 of this article. The *aCH* component poorly fits the 3–5 eV region (see the residual of the fit plotted in Figure S16, Supporting Information). This could be due to the grouping and simplifying a mix of amorphous carbon species, such as surface methyl groups from slurry casting and air storage, inter‐graphitic amorphous carbon, and tetrahedral amorphous carbon, into the *aCH* component. The *aCH*:*HOPG*_total_ area ratio is 1.19 and no *sp* ^3^ component was fitted which indicates a highly graphitic carbon in the blue region. In the green region, the *aCH*:*HOPG*_total_ area ratio is 6.32. This indicates the green region is related to polymeric amorphous carbon.

The CSEHI, color segmentation, and SE spectrum fitting workflow quickly mapped and identified regions of graphite active material and carbon binder domain in the field of view. The segmentation indicates from the plan‐view that 21% of the graphite is uncoated. The SE spectrum fitting of the binder domain successfully identified the oxygen functionality in the binder domain, originating from CMC binder.

### 5.3 Mechanochemically Functionalized Carbon Black

#### 5.3.1 Material Description and Previous Findings by Standard Methods

The CBs provided for this study were previously produced and characterized by Kiani et al.^[^ ^43^ ^]^ The experimental conditions of ball milling are given in Table S2 (Supporting Information). The five CB samples were: as‐received CB and CB ball milled at room temperature without solvent for a total of 1, 5, 9, and 11 h. Kiani et al. used XPS to characterize the surface of the CB and FTIR to detect the functional groups produced by ball milling. The FTIR absorbance spectra showed that by 5 h ball milling time there were functional groups which are a product of oxidation: peroxide, epoxide, ether, alcohol and carbonyl at 607, 896, 1057, 1163, and 1700 cm^−1^ respectively.^[^ ^43^ ^]^ With further ball milling there was increased carbonyl and alcohol group absorbance and a reduction in peroxide and epoxide absorbance. After 5 h ball milling time there was a larger absorbance by vinyl‐ether stretching (1220‐1225 cm^−1^).

XPS spectrum fitting in the C1s region (280–295 eV) by Kiani et al. used five components for: sp^2^, C─O, C ═ O, O–C ═ O and shake‐up. The oxygen and carbon atomic fractions found by fitting these components were used to obtain an O/C ratio for the untreated CB and each subsequent ball milling time (Figure 18b).

#### 5.3.2 Characterization and New Insights to Ball Milling Process

**Figure 17:**
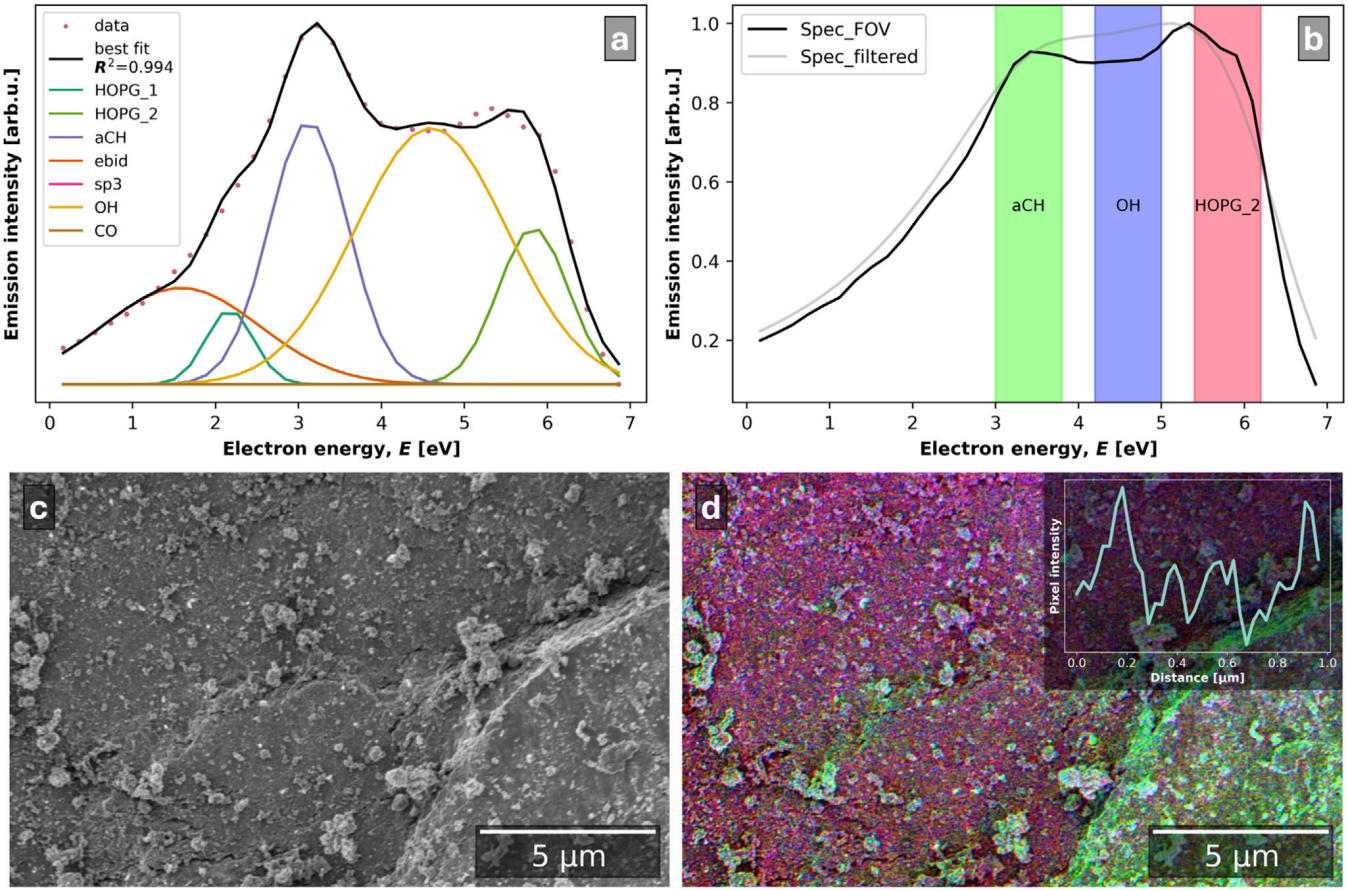
a) Average SE spectrum of CB‐5 h with Gaussian components HOPG_1, HOPG_2, aCH, “ebid,” sp^3^, OH, and CO. b) SE spectrum of CB‐5 h area shown in (c,d) with energy ranges used to color the image (d) which are: R, “HOPG_2,” [5.4–6.2 eV]; G, “aCH,” [3–3.8 eV]; B, “OH,” [4.2–5.0 eV]. c) SE image of an area of the surface of CB‐5 h and d) corresponding color image produced using the ranges in (b) with inset line profile of pixel intensity (region of line profile shown in Figure S18, Supporting Information).

CB powder specimens were prepared for SEHI as in Nohl et al.^[^ ^13^ ^]^ Average spectra from SEHI volumes covering regions of 20 µm HFW and SE energies (0–7 eV) were produced from surfaces of each of the CB samples (1 h, etc.). A fitting model was developed for the SE spectra from functionalized CBs which included components for *OH* and *CO* oxidation products, as identified by FTIR spectroscopy and XPS.^[^ ^43^ ^]^ The initial peak center values given for *OH* and *CO* were 4.60 and 5.2 eV respectively. The full model initial parameters are found in Section S3.4.1 (Supporting Information). The peak centers for *OH* and *CO* components were reported by Farr et al. from studies of polymer films: polypropylene (PP), air plasma treated PP, nylon‐6 and polycaprolactone (PCL) and aged HOPG as well as in phenolic resin systems.^[^ ^10^, ^11^ ^]^ **Figure** **17**a shows the average SE spectrum of the CB‐5 h material with components which result from fitting the CB SE spectrum model.

The positions of fitted component peak centers the SE spectrum in ±0.4 eV resulted in the energy ranges of 5.4–6.2 eV for sp^2^; 3.0–3.8 eV for aCH; 4.2–5.0 eV for OH. These ranges are overlaid on Figure 17b which originates from the image region in Figure 17c. The color ranges give the color image in Figure 17d. A CSEHI image produced by an alternative color assignment order is provided in Figure S17 (Supporting Information) to aid readers with difficulty distinguishing red‐green color contrast. The color map shows *HOPG_2* (i.e., sp^2^) as red, *aCH* as green and *OH* as blue. The underlying surface appears sp^2^ in character. Atop this, there are particles of amorphous hydrogenated carbon character. A crack or “terrace” in the bulk particle surface also has edges of amorphous hydrogenated carbon. The hydroxy functionality appears generally dispersed along with sp^2^ resulting in a purple mix. The inset line profile of pixel intensity is along a 1 µm length, indicating the size of satellite nanoparticles (line profile region shown in Figure S18, Supporting Information).

**Figure 18:**
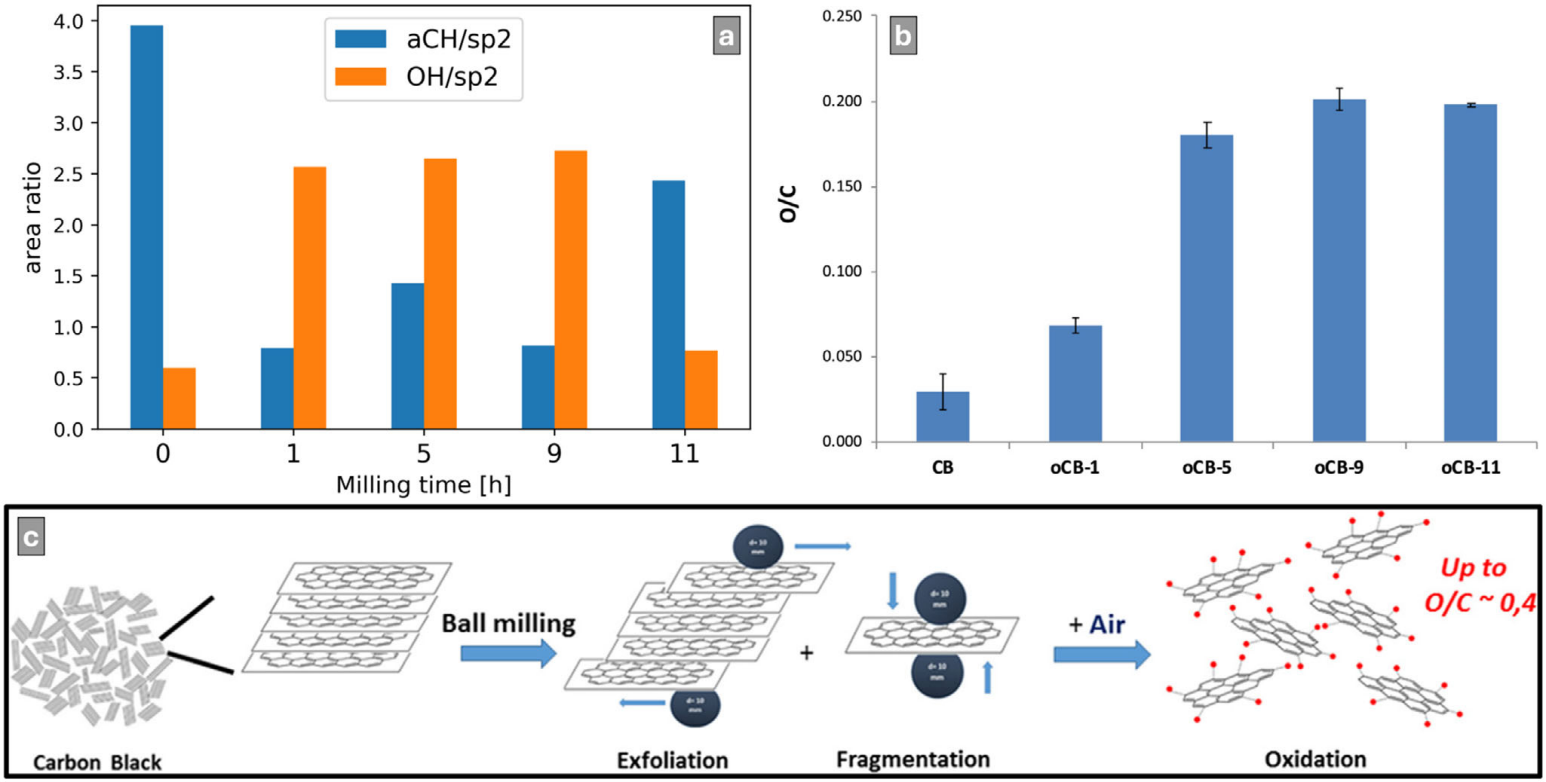
a) Bar plot of area ratios aCH/sp^2^ and OH/sp^2^ versus milling time. b) O/C atomic ratio from XPS C 1s scan region. Reproduced from Kiani et al., ACS Sustainable Chem. Eng. (2022), under the terms of the CC BY 4.0 license.^[^ ^43^ ^]^ c) Schematic of carbon black exfoliation, fragmentation and oxidation by ball milling in air. Reproduced from Kiani et al., ACS Sustainable Chem. Eng. (2022) under the terms of the CC BY 4.0 license.^[^ ^43^ ^]^

Ratios of peak heights for *aCH* (hydrogenated amorphous carbon) and *OH* functionalized carbon to sp^2^‐hybridized graphitic carbon were calculated throughout the ball milling series (**Figure** **18**a). The *aCH*:*sp* ^2^ ratio (where *sp* ^2^ is the sum of *HOPG_1* and *HOPG_2* peak areas) decreases from a maximum of 3.95 at 0 h ball milling to a minimum of 0.79 at 1 h, then decreases to 0.81 at 9 h before increasing to 2.42 at 11 h.

The *OH*:*sp* ^2^ ratio increases from a minimum of 0.60 at 0 h to 2.57 at 1 h, 2.65 at 5 h and a maximum of 2.72 at 9 h, before decreasing to 0.76 at 11 h.

The combined minima of *aCH*+*OH*/*sp* ^2^ are at 1 h and 9 h. This is where *sp* ^2^ is at maximum peak height. We therefore propose that the first hour of ball‐milling exfoliates and produces fresh sp^2^ surfaces. Surfaces oxidize by 5 h and slightly increase up to 9 h. Further milling appears to return the surface to an amorphous carbon character by 11 h. This sequence of surface functionalities generally supports the model in Figure 17c, where exfoliation is followed by fragmentation then by oxidation. Perhaps the cycle repeats from 11 h onward, with newly exfoliated sp^2^ character surfaces.

SEHI added to the understanding of the CB material by analyzing the local chemistry of “satellite” nanoparticles which had amorphous hydrogenated carbon and oxidized character. Also visible were amorphous hydrogenated carbon edges from cracking of the graphitic bulk. Local characterization related surface chemistry to morphology and supports the model of exfoliation then oxidation. Results from SEHI might suggest that an amorphization proceeds oxidation. The nano‐FTIR measurements of the HOPG surface post air exposure (Section S2.4, Supporting Information) showed oxidation in all points, including those with EBID treatment (expected to produce amorphous hydrogenated carbon), indicating that amorphous hydrogenated carbon will oxidize further in air to oxygen containing functional groups. XPS fitting with a component for non‐graphitic C─C bonding could be used to provide evidence for this oxidation route.

## 6 Limitations and Opportunities

### 6.1 Limitations

Raman spectroscopy, due to its larger information depths and thus reduced surface sensitivity, produces spectra least prone to exhibit effects of EBID. However, EBID can affect Raman spectra due to electric field effects.^[^ ^86^ ^]^

Although XPS is carried out under UHV conditions, adsorption of atmospheric hydrocarbons onto surfaces prepared in the typical laboratory environment before introduction into UHV can result in the presence of a C 1s signal. The C1s is intensity related to the amount of contamination.^[^ ^87^ ^]^ Such exposure can also effect SEHI and the exact composition of EBID.

As shown in Figure S3a,b (Supporting Information) it is apparent that EBID can be affected by the microstructure of the sample. This can be understood through changes to diffusion processes in a flat and structured material. Similarly, the exact nature of EBID depends on the instrumentation used and cleaning methods applied as described in Section 4.2. Thus, measurements susceptible to EBID cannot be generalized but should be carefully evaluated for each instrument and cleaning protocol to allow for reliable interpretation of collected data. The peak fitting introduced here can be used to help with such evaluation as demonstrated in Section 4.2.

Moisture from the FIB‐vacuum chamber deposited on the surface and forming oxides and atomic hydrogen^[^ ^33^ ^]^ could be present if cleaning procedures such as plasma cleaning of the chamber outlined in Section 4.3 cannot be implemented. This could also contribute to differences between SEHI spectra collected from the same sample in different instruments.

The complexity of SEHI data is a challenge we hope to begin to tackle in this work. However further studies on the effect of fitting parameters and background correction would be beneficial for widespread application and moving towards the wide range of applications outlined in the opportunity section.

The energy region for SEHI as in the configuration demonstrated here only allows access to the energy region up to 7 eV. SEs with higher energies cannot be accessed reliably but could contain valuable information to validate peak identification.

The sensitivity to different types of aCH is not tested. AES has been suggested to be more sensitive to local ordering and appears useful in discriminating between various forms of amorphous carbon.^[^ ^49^ ^]^

### 6.2 Opportunities

SEHI combines surface sensitivity of XPS with lateral resolution similar or better than Raman spectroscopy and imaging. SEHI can therefore reveal lateral variations in carbon bonding on the nanoscale as demonstrated in Figures 7, 15, and 17d. This then enables for example estimates of available area of active material as demonstrated in Figure 16. This is important for devices containing a mixture of carbon materials with different functions.

A wide variety of carbon nanomaterials has demonstrated high electrocatalytic performance across systems and devices for clean energy generation, storage, green chemistry or environmental remediation. It requires controlled defects via advanced nanotechnology but needs to be coupled with advanced characterization techniques.^[^ ^88^ ^]^ For example, defects in carbon based electro‐catalysts experience dynamic structural transformation that should not be ignored.^[^ ^89^ ^]^ SEHI could enable the mapping of defect distributions in components such as gas diffusion layers or catalyst supports in fuel cells, provided suitably high‐resolution SEHI data are collected and analyzed with a sufficient number of relevant peaks.

Carbon is of vital importance to the behavior of metallic systems. In steels, it is a fundamental addition that drives the development of the microstructure and key phase transformations, notably influencing hardenability and strength.^[^ ^90^ ^]^ In many specialist steels, complex carbides are intentionally precipitated during heat treatment to tailor performance to the required application. However, when in service, particularly at high temperature, these carbide distributions can be adversely affected, resulting in a degradation of material performance over time. It is therefore critical, to be able to reliably analyze these carbides during metallurgical investigations. Some of these carbide compositions and distributions can be challenging.^[^ ^91^ ^]^ A standard process for analysis may include optical microscopy of an etched sample for grain size determination followed by backscattered electron imaging for secondary phase imaging. However, with many phases, the contrast between the secondary phase and substrate is small or inconsistent, meaning that focused ion beam imaging is used for quantification of large carbides. It was recently shown that using this method, there is a possibility of misidentification of some phases which can be rectified through the use of detection of the secondary electron signal using an in column detector, with some degree of energy filtering.^[^ ^92^ ^]^ Therefore, the use of SEHI to further enhance contrast and to benefit from the good spatial resolution and damage free electron beam imaging, could offer an opportunity to distinguish and map carbides to the nanoscale provided relevant reference data are available and can inform analysis through relevant peak fitting models.

Beyond steels, various materials utilize carbon as a grain boundary strengthening element; both the segregation of carbon to grain boundaries and carbide formation are fundamental to delivering component performance. Carbon is a common grain boundary strengthening element in superalloys and typically added in the range of 0.06–0.15 wt%.^[^ ^93^ ^]^ C─N co‐doping enhances phase stability and mechanical properties of the high entropy alloys due to nano‐sized carbonitrides.^[^ ^93^ ^]^

In addition to intentional carbon additions to drive strengthening and phase transformations, carbon is a ubiquitous contaminant in metallic systems, especially in powder metallurgy. For example, microstructural analysis of high entropy alloy powders obtained through mechanical alloying by ball milling exhibited substantial carbon contaminant phases observed by SEM, and sufficiently high to be detected by EDX.^[^ ^94^ ^]^ As even traces of carbon can affect phase stability and mechanical properties, detecting trace amounts of carbon down to the nanoscale and understanding its role is therefore of critical importance in metallic materials.

SEHI can be applied to metals such as lithium,^[^ ^12^ ^]^ transition metal oxides, including those used in pharmaceutical compounds.^[^ ^95^ ^]^ The common challenge with carbon materials is the complexity of the SE spectra. For each of these groups for materials, peak fitting models will need to be developed using a methodology reflecting the methods for data acquisition and peak fitting introduced here for carbon materials. Depending on the complexity of specific materials systems, high resolution spectra and models with larger numbers of peaks might need to be developed.

The use of different spectral regions to reveal defects in semiconductors such as GaN was also demonstrated previously but could be much improved by SEHI.^[^ ^96^ ^]^ For semiconductors SE spectroscopy was used as early 1967 as to reveal differently doped areas in silicon.^[^ ^97^ ^]^ SEM dopant contrast is still of technological interest, especially for doping in nanostructured materials.^[^ ^98^ ^]^ It was reported that graphitic carbon built up could affect dopant mapping.^[^ ^99^ ^]^ The SE spectroscopy and SEHI analysis methods proposed here could be used to investigate this further for instance to gain information about the carbon bonding and how this might affect dopant mapping in different instruments.

For some of the more complex materials systems, the ability to carry out survey scans that could include other peaks such as Auger peaks and the inelastically back scattered peak would be highly beneficial to inform component fitting, background subtraction and cross‐validation. This could be made possible using add‐on spectrometers.

Path to realizing opportunities:

1. Add‐on spectrometers that allow for whole spectrum as well as high resolution SE spectra collections could be used to overcome some of the current limitations. It could enable accurate modeling for validations and prediction of optimum experimental conditions and background correction approaches.
2. Add‐on spectrometers could also be used to check/correct calibration of different SEHI cable instruments.
3. A database containing spectra alongside instrument information, calibration, and experiential collection conditions would enable a more comprehensive evaluation of the ultimate limits of FIB‐SEM‐SEHI which is why the data used in this work can be accessed through https://doi.org/10.15131/shef.data.28228484.
4. An extensive database would also enable Machine Learning and Artificial Intelligence methods to further interrogate the complexity of SEHI data considering reliability and supporting transfer to other materials systems.

## 7 Conclusion 

The strength of SEHI lies in the visualization of carbon order/disorder regions down to the nanoscale connecting nano to microscale surface images and spectroscopy within FIB‐SEMs. This was demonstrated on example application materials for which characterization has been a longstanding challenge. Graphitic and disordered carbon was mapped at the length scale of primary and secondary particles of lithium iron phosphate. Regions of binder were differentiated from graphitic active material in the cast graphite anode from a LIB. CB surface chemistry was associated with nanoscale features resulting from mechanochemical processing. SEHI provided direct evidence of exfoliation and fragmentation and highlighted structural differences in fragments and exfoliated areas. SEHI can be used to determine when ball milling should be terminated. These findings were compared to a previous spatially averaged XPS analysis of the CBs and delivered consistent findings with the model of exfoliation, fragmentation and oxidation which was developed but could not look at satellite particles and their surface chemical differences.

Key to such application was a peak fitting model for SE spectra of carbon which was inspired and informed by standard spectroscopy techniques, XPS and Raman. For the first time, the carbon SE‐spectrum peak fitting models were used to establish peak area and position in SE spectra of HOPG and disordered carbons enabling a sound framework for the selection of energy bands used for visualization. Using a peak fitting model for disordered graphitic carbons which included information from HOPG reference material, peak area ratios such as aCH/sp^2^ were reported as part of a qualitative comparison of SE‐spectra of different carbon materials obtained in the same FIB‐SEM. The model also paved the way for a comparison of SEHI analyses in different FIB‐SEMs. It highlighted variation between FIB‐SEMs instruments. Regardless of such differences, components in SE spectrum models exhibited sensitivity to sp^2^ and sp^3^ carbon bonding and disordered and amorphous carbons.

Systematic collection and analysis of HOPG gave new insights into the extent and nature of EBID contamination under different LV‐SEM vacuum, temperature and chamber pre‐cleaning conditions to guide potential SEHI users. It also enabled a comparison of effectiveness of these interventions. Plasma cleaning had the greatest effect in reducing EBID contamination (provided vacuum levels are in an acceptable range). While we have established experimental workflows and related software tools for the FIB‐SEM community to access SEHI analysis, and to provide insight into graphitic and disordered carbon materials without the need for additional hardware, these workflows are expected to be transferable and of benefit to a much wider range of technologically important materials systems. Applications such as next generation metal alloy development, next generation semiconductors materials and devices, alongside next generation energy storage and harvesting devices could benefit. We expect that the methodology presented in this work can guide and support transfer and correct implementation of SEHI in future technology developments as highlighted in the opportunities section.

## Conflict of Interest

The authors declare no conflict of interest.

## Author Contributions

J.F.N.: Conceptualization, Data curation, Formal analysis, Investigation, Methodology, Project administration, Software, Validation, Visualization, Writing—original draft, Writing—review and editing. Ni.T.H.F.: Formal analysis, Investigation, Methodology, Writing—review and editing. M.R.A.: Investigation, Methodology. A.J.K.: Data curation, Formal analysis, Investigation, Methodology, Writing—review and editing. G.M.H.: Data curation, Formal analysis, Investigation, Methodology, Writing—review and editing. J.Z.: Data curation. S.R.: Data curation, Formal analysis, Investigation. S.M.: Data curation, Formal analysis, Investigation. S.M.: Data curation, Investigation. T.M.: Data curation, Formal analysis, Investigation. C.W.: Data curation, Formal analysis, Investigation. A.I.T.: Funding acquisition, Project administration, Resources. F.M.: Funding acquisition, Project administration, Resources. Z.P.: Funding acquisition, Project administration, Resources. S.T.: Funding acquisition, Project administration, Resources. A.P.: Funding acquisition, Project administration, Resources. N.L.F.: Funding acquisition, Project administration, Resources. N.H.: Funding acquisition, Project administration, Resources. M.A.E.J.: Funding acquisition, Project administration, Resources. L.S.M.: Funding acquisition, Project administration, Resources. N.R.‐M.: Project administration, Supervision, Writing—review and editing. S.A.C.: Funding acquisition, Project administration, Resources, Supervision, Writing—review and editing. C.R.: Conceptualisation, Funding acquisition, Methodology, Project administration, Resources, Supervision, Writing—review and editing.

## Supporting information



Supporting Information

## Data Availability

Data for “Secondary electron hyperspectral imaging of carbons: New insights and good practice guide” are available at https://doi.org/10.15131/shef.data.28228484.

## References

[1] J. Asenbauer , T. Eisenmann , M. Kuenzel , A. Kazzazi , Z. Chen , D. Bresser , Sustainable Energy Fuels 2020, 4, 5387.

[2] Y.‐C. Chang , C.‐T. Peng , I.‐M. Hung , J. Mater. Sci. 2014, 49, 6907.

[3] M. Kumar , X. Xiong , Z. Wan , Y. Sun , D. C. Tsang , J. Gupta , B. Gao , X. Cao , J. Tang , Y. S. Ok , Bioresour. Technol. 2020, 312, 123613. 32513509 10.1016/j.biortech.2020.123613

[4] Z. Shen , X. Gao , S. Zhang , Z. Li , H. Zhao , Appl. Surf. Sci. 2022, 606, 154931.

[5] L. Larbi , B. Larhrib , L. Madec , C. Vaulot , L. Monconduit , C. M Ghimbeu , ACS Appl. Energy Mater. 2024, 7, 3378.

[6] H. Shin , S. Jeong , J. Hong , E. Wi , E. Park , S. I. Yang , J.‐T. Kwon , H. Lee , J. Lee , Y. Kim , Environ. Pollut. 2023, 330, 121787. 37156438 10.1016/j.envpol.2023.121787

[7] R. R. Yuan , Y. Dong , R. Hou , L. Shang , J. Zhang , S. Zhang , X. Chen , H. Song , Chem. Eng. J. 2023, 454, 140418.

[8] R. C. Masters , A. J. Pearson , T. S. Glen , F.‐C. Sasam , L. Li , M. Dapor , A. M. Donald , D. G. Lidzey , C. Rodenburg , Nat. Commun. 2015, 6, 6928. 25906738 10.1038/ncomms7928 PMC4423221

[9] N. Stehling , R. Masters , Y. Zhou , R. O'Connell , C. Holland , H. Zhang , C. Rodenburg , MRS Commun. 2018, 8, 226.

[10] N. Farr , J. Thanarak , J. Schäfer , A. Quade , F. Claeyssens , N. Green , C. Rodenburg , Adv. Sci. 2021, 8, 2003762. 10.1002/advs.202003762 PMC7887591 33643809

[11] N. T. H. Farr , S. F. Hamad , E. Gray , C. M. Magazzeni , F. Longman , D. E. J. Armstrong , J. P. Foreman , F. Claeyssens , N. H. Green , C. Rodenburg , Polym. Chem. 2021, 12, 177.

[12] J. F. Nohl , N. T. Farr , Y. Sun , G. M. Hughes , N. Stehling , J. Zhang , F. Longman , G. Ives , Z. Pokorná , F. Mika , V. Kumar , L. Mihaylova , C. Holland , S. A. Cussen , C. Rodenburg , Mater. Today Adv. 2023, 19, 100413.

[13] J. F. Nohl , N. T. Farr , Y. Sun , G. M. Hughes , S. A. Cussen , C. Rodenburg , Micron 2022, 156, 103234. 35325668 10.1016/j.micron.2022.103234

[14] C. Walker , M. M. ElGomati , A. M. D. Assa'd , M. Zadražil , Scanning 2008, 30, 365. 18661504 10.1002/sca.20124

[15] M. M. El‐Gomati , C. G. H. Walker , A. M. D. Assa'd , M. Zadražil , Scanning 2008, 30, 2. 18302216 10.1002/sca.20091

[16] I. Konvalina , F. Mika , S. Krátký , E. M Mikmeková , I. Müllerová , Materials 2019, 12, 2307. 31330942 10.3390/ma12142307 PMC6679021

[17] S. Ono , K. Kanaya , J. Phys. D: Appl. Phys. 1979, 12, 619.

[18] Y. B. Zou , S. F. Mao , B. Da , Z. J. Ding , J. Appl. Phys. 2016, 120, 235102.

[19] J. Park , J. Kim , H. Kwon , Bull. Korean Chem. Soc. 2020, 41, 34.

[20] B. Boldrini , E. Ostertag , K. Rebner , D. Oelkrug , Anal. Bioanal. Chem. 2021, 413, 7093. 34599394 10.1007/s00216-021-03678-w PMC8589783

[21] S. Tanuma , C. J. Powell , D. R. Penn , Surf. Interface Anal. 2011, 43, 689

[22] T. Taubner , R. Hillenbrand , F. Keilmann , J. Microsc. 2003, 210, 311. 12787105 10.1046/j.1365-2818.2003.01164.x

[23] F. Huth , A. Govyadinov , S. Amarie , W. Nuansing , F. Keilmann , R. Hillenbrand , Nano Lett. 2012, 12, 3973. 22703339 10.1021/nl301159v

[24] L. Mester , A. A. Govyadinov , S. Chen , M. Goikoetxea , R. Hillenbrand , Nat. Commun. 2020, 11, 3359. 32620874 10.1038/s41467-020-17034-6 PMC7335173

[25] M. Lewin , B. Hauer , M. Bornhöfft , L. Jung , J. Benke , A.‐K. U. Michel , J. Mayer , M. Wuttig , T. Taubner , Appl. Phys. Lett. 2015, 107, 151902.

[26] P. R. Griffiths , J. A. de Haseth , Introduction to Vibrational Spectroscopy, John Wiley & Sons, Ltd, Chichester, West Sussex, United Kingdom, Ch. 1, 2007, p. 1.

[27] J. J. Friel , C. E. Lyman , Microsc. Microanal. 2006, 12, 2. 17481338 10.1017/S1431927606060211

[28] J. B. Kortright , A. C. Thompson , X‐ray Data Booklet, Center for X‐ray Optics and Advanced Light Source, Lawrence Berkeley National Laboratory, USA 2001, p. 35.

[29] M. Haider , Hartel , H. Müller , S. Uhlemann , J. Zach , Microsc. Microanal. 2010, 16, 393. 20598203 10.1017/S1431927610013498

[30] D. J. Smith , Micron 2012, 43, 504.

[31] R. Egerton , M. Watanabe , Micron 2022, 160, 103304. 35704972 10.1016/j.micron.2022.103304

[32] P. Soni , in *Proceedings of the Microscience Microscopy Congress 2023 incorporating EMAG*, ser. mmc2023. Royal Microscopical Society, Oxford, UK, 2023.

[33] M. Konno , T. Ogashiwa , T. Sunaoshi , Y. Orai , M. Sato , Ultramicroscopy 2014, 145, 28. 24290787 10.1016/j.ultramic.2013.09.001

[34] A. C. Ferrari , Solid State Commun. 2007, 143, 47.

[35] A. C. Ferrari , J. Robertson , Philos. Trans. R. Soc., A 2004, 362, 2477. 10.1098/rsta.2004.1452 15482988

[36] C. Castiglioni , M. Tommasini , G. Zerbi , Philos. Trans. R. Soc., A 2004, 362, 2425. 10.1098/rsta.2004.1448 15482986

[37] A. Milani , M. Tommasini , V. Russo , A. Li Bassi , A. Lucotti , F. Cataldo , C. S. Casari , Beilstein J. Nanotechnol. 2015, 6, 480. 25821689 10.3762/bjnano.6.49 PMC4362090

[38] A. Ferrari , J. Robertson , Phys. Rev. B 1999, 61, 14095.

[39] J. Shi , X. Shi , Z. Sun , S. Lau , B. Tay , H. Tan , Diamond Relat. Mater. 2001, 10, 76.

[40] D. S. Knight , W. B. White , J. Mater. Res. 1989, 4, 385.

[41] R. Ramesham , T. Roppel , R. Johnson , J. Chang , Thin Solid Films 1992, 212, 96.

[42] K. J. Abrams , M. Dapor , N. Stehling , M. Azzolini , S. J. Kyle , J. Schäfer , A. Quade , F. Mika , S. Kratky , Z. Pokorna , I. Konvalina , D. Mehta , K. Black , C. Rodenburg , Adv. Sci. 2019, 6, 1900719. 10.1002/advs.201900719 PMC6774015 31592411

[43] A. Kiani , M. R. Acocella , V. Granata , E. Mazzotta , C. Malitesta , G. Guerra , ACS Sustainable Chem. Eng. 2022, 10, 16019.

[44] N. Ohtake , M. Hiratsuka , K. Kanda , H. Akasaka , M. Tsujioka , K. Hirakuri , A. Hirata , T. Ohana , H. Inaba , M. Kano , H. Saitoh , Materials 2021, 14, 315. 33435425 10.3390/ma14020315 PMC7827220

[45] L. Zhang , X. Wei , Y. Lin , F. Wang , Carbon 2015, 94, 202.

[46] M. Hugenschmidt , K. Adrion , A. Marx , E. Müller , D. Gerthsen , Microsc. Microanal. 2023, 29, 219.

[47] E. Materna Mikmeková , I. Müllerová , L. Frank , A. Paták , J. Polčák , S. Sluyterman , M. Lejeune , I. Konvalina , J. Electron Spectrosc. Relat. Phenom. 2020, 241, 146873.

[48] D. Lu , K. Goto , B. Da , J. Liu , H. Yoshikawa , S. Tanuma , Z. Ding , J. Electron Spectrosc. Relat. Phenom. 2021, 250, 147086.

[49] B. Lang , Surf. Sci. 1979, 80, 38.

[50] N. Farr , S. Pashneh‐Tala , N. Stehling , F. Claeyssens , N. Green , C. Rodenburg , Macromol. Rapid Commun. 2019, 41, 2070006. 10.1002/marc.201900484 31859420

[51] M. S. Chung , T. E. Everhart , J. Appl. Phys. 1974, 45, 707.

[52] W. Han , M. Zheng , A. Banerjee , Y. Z. Luo , L. Shen , A. Khursheed , Sci. Rep. 2020, 10, 22144. 33335154 10.1038/s41598-020-78973-0 PMC7746715

[53] A. Khursheed , Scanning Electron Microscope Optics, Spectrometers, World Scientific, NUS, Singapore, 2010, p. 416.

[54] I. Krainsky , V. Asnin , A. Petukhov , Secondary Electron Emission Spectroscopy of Diamond Surfaces, ser. NASA technical paper, NASA Glenn Research Center, 1999.

[55] Z. Li , Y. Wang , A. Kozbial , G. Shenoy , F. Zhou , R. McGinley , Ireland , B. Morganstein , A. Kunkel , S. P. Surwade , L. Li , H. Liu , Nat. Mater. 2013, 12, 925. 23872731 10.1038/nmat3709

[56] A. Bird , L. Yang , G. Wu , B. Inkson , Wear 2023, 530–531, 205034.

[57] S. Marchesini , Turner , K. R. Paton , B. P. Reed , B. Brennan , K. Koziol , A. J. Pollard , Carbon 2020, 167, 585.

[58] F. Rozpoch , J. Patyk , J. Stankowski , Acta Phys. Pol. A 2007, 112, 557.

[59] R. M. Dey , M. Pandey , D. Bhattacharyya , D. S. Patil , S. K. Kulkarni , Bull. Mater. Sci. 2007, 30, 541.

[60] FEI Company , Helios NanoLab 450/450 S/450 ML/650/600i User Operation Manual, 2nd ed., FEI Company, Hillsboro, OR, 2011.

[61] P. Kazemian , S. Mentink , C. Rodenburg , C. Humphreys , Ultramicroscopy 2007, 107, 140. 16872746 10.1016/j.ultramic.2006.06.003

[62] T. Burnett , R. Kelley , B. Winiarski , L. Contreras , M. Daly , A. Gholinia , M. Burke , Withers , Ultramicroscopy 2016, 161, 119. 26683814 10.1016/j.ultramic.2015.11.001

[63] A. Suri , A. Pratt , S. Tear , C. Walker , M. El‐Gomati , J. Microsc. 2020, 279, 207. 31985065 10.1111/jmi.12867 PMC8597398

[64] L. Frank , R. Steklý , M. Zadražil , M. M. El‐Gomati , I. Müllerová , Microchim. Acta 2000, 132, 179.

[65] A. Vladar , M. Postek , Microsc. Microanal. 2005, 11, 764.

[66] N. T. H. Farr , G. M. Hughes , C. Rodenburg , Materials 2021, 14, 3034. 34199625 10.3390/ma14113034 PMC8199708

[67] K.‐H. Müller , Ph.D. dissertation, 1971.

[68] N. A. Stehling , Ph.D. dissertation, University of Sheffield, 2019.

[69] P. Zhang , L. Zhang , Int. J. Therm. Sci. 2021, 161, 106714.

[70] W. Li , D. C. Joy , J. Vac. Sci. Technol., A 2006, 24, 431.

[71] P. Hirsch , M. Kässens , M. Püttmann , L. Reimer , Scanning 1994, 16, 101.

[72] E. Müller , A. J. Schwartz , Y. M. Eggeler , BIO Web Conf. 129, 22004, 2024.

[73] R. O'Connell , Ph.D. dissertation, Trinity College Dublin, 2018.

[74] J. Nohl , N. Farr , N. Stehling , J. Zhang , F. Longman , G. Ives , L. Mihaylova , C. Holland , C. Rodenburg , CSEHI_app 1.0, 2023.

[75] A. Schwartz , E. Müller , Y. Eggeler , BIO Web Conf. 2024, 129, 22041.

[76] F. Degen , M. Winter , D. Bendig , J. Tübke , Nat. Energy 2023, 8, 1284.

[77] T. Satyavani , A. Srinivas Kumar , Subba Rao , Eng. Sci. Technol. Int. J. 2016, 19, 178.

[78] C.‐Z. Lu , G. T.‐K. Fey , H.‐M. Kao , J. Power Sources 2009, 189, 155.

[79] J. V. Laveda , B. Johnston , G. W. Paterson , J. Baker , M. G. Tucker , H. Y. Playford , K. M. Ø. Jensen , S. J. L. Billinge , S. A. Corr , J. Mater. Chem. A 2018, 6, 127.

[80] A. A. Ryabin , D. V. Pelegov , Appl. Spectrosc. 2022, 76, 1335. 35484849 10.1177/00037028221100843

[81] Z. Jinli , W. Jiao , L. Yuanyuan , N. Ning , G. Junjie , Y. Feng , L. Wei , J. Mater. Chem. A 2015, 3, 2043.

[82] W. Paraguassu , T. C. Freire , V. Lemos , S. M. Lala , L. A. Montoro , J. M. Rosolen , J. Raman Spectrosc. 2005, 36, 213.

[83] R. Wang , L. Feng , W. Yang , Y. Zhang , Y. Zhang , W. Bai , B. Liu , W. Zhang , Y. Chuan , Z. Zheng , H. Guan , Nanoscale Res. Lett. 2017, 12, 575. 29086045 10.1186/s11671-017-2348-6 PMC5662525

[84] M. J. Jolley , T. S. Pathan , C. Jenkins , M. J. Loveridge , J. Appl. Polym. Sci. 2023, 141, 55135.

[85] M. Ryu , Y.‐K. Hong , S.‐Y. Lee , J. H. Park , Nat. Commun. 2023, 14, 1316. 36899006 10.1038/s41467-023-37009-7 PMC10006413

[86] D. Lau , A. E. Hughes , T. H. Muster , T. J. Davis , A. M. Glenn , Microsc. Microanal. 2009, 16, 13. 20030911 10.1017/S1431927609991206

[87] G. C. Smith , J. Electron Spectrosc. Relat.Phenom. 2005, 148, 21.

[88] Q. Zhai , H. Huang , T. Lawson , Z. Xia , Giusto , M. Antonietti , M. Jaroniec , M. Chhowalla , J. Baek , Y. Liu , S. Qiao , L. Dai , Adv. Mater. 2024, 36, 2405664. 10.1002/adma.202405664 39049808

[89] Q. Wu , H. Zou , X. Mao , J. He , Y. Shi , S. Chen , X. Yan , L. Wu , C. Lang , B. Zhang , L. Song , X. Wang , A. Du , Q. Li , Y. Jia , J. Chen , X. Yao , Nat. Commun. 2023, 14, 6275. 37805502 10.1038/s41467-023-41947-7 PMC10560253

[90] G. Krauss , Steels: Processing, Structure, and Performance, 2nd ed. ASM International, Almere, the Netherlands, 2015.

[91] C. Rodenburg , W. M. Rainforth , J. Appl. Phys. 2006, 100, 114902.

[92] R. G. Byrne , R. J. McGladdery , Z. Zhou , R. C. Thomson , S. S. Doak , M. A. Jepson , Mater. Charact. 2020, 165, 110356.

[93] W. Zhang , D. Yan , W. Lu , Z. Li , J. Alloys Compd. 2020, 831, 154799.

[94] I. Moravcik , A. Kubicek , L. Moravcikova‐Gouvea , O. Adam , V. Kana , V. Pouchly , A. Zadera , I. Dlouhy , Metals 2020, 10, 1186.

[95] S. Micklethwaite , R. Basnet , J. Ma , D. Hopper , J. Nohl , N. Farr , C. Rodenburg , N. Hondow , BIO Web Conf. 2024, 129, 06027.

[96] C. Schönjahn , C. J. Humphreys , M. Glick , J. Appl. Phys. 2002, 92, 7667.

[97] T. Chang , W. Nixon , Solid‐State Electron. 1967, 10, 701.

[98] Y. Zhang , G. Scardera , S. Wang , M. Abbott , D. Payne , B. Hoex , Adv. Mater. Technol. 2022, 8, 2200737.

[99] M. El‐Gomati , F. Zaggout , H. Jayacody , S. Tear , K. Wilson , Surf. Interface Anal. 2005, 37, 901.

