# Supplementary material for: Secondary Electron Hyperspectral Imaging of Carbons: New Insights and Good Practice Guide
Source: Adv Sci (Weinh). 2025 Jun 17;12(29):e01907. doi: 10.1002/advs.202501907 (PMC12362828; doi:10.1002/advs.202501907)
Supplement: Supplementary file 1 — Supporting Information [file ADVS-12-e01907-s001.docx]

Supporting Information

Secondary electron hyperspectral imaging of carbons: New insights and good practice guide

James F. Nohl, Nicholas TH. Farr, Maria Rosaria Acocella, Alexander J. Knight, Gareth M. Hughes, Jingqiong Zhang, Stuart Robertson, Stuart Micklethwaite, Sean Murphy, Tereza Motlová, Christopher Walker, Alexander I. Tartakovskii, Filip Mika, Zuzana Pokorná, Steve Tear, Andrew Pratt, Nancy L. Ford, Nicole Hondow, Mark AE. Jepson, Lyudmila S. Mihaylova, Nik Reeves‑McLaren, Serena A. Cussen, Cornelia Rodenburg*

# **Bessel-box detector SE spectra**


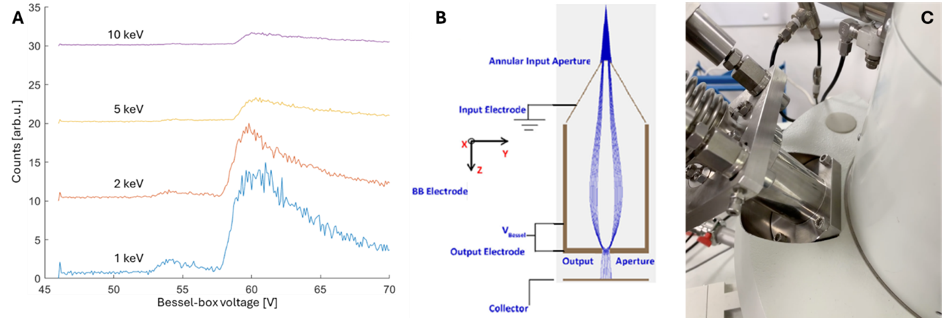


**Figure S1.** (a) SE spectra of exfoliated HOPG acquired with incident electron energies: 1 keV (light blue), 2 keV (red), 5 keV (yellow) and 10 keV (violet). The sample was biased by -49.6 V and that the spectra are offset for clarity. (b) Schematic of the Bessel box showing that for a given set of voltages the BB focuses a narrow energy range of electrons at the output aperture before they are collected [63]. Reproduced under CC BY 4.0 license^[[1]](#footnote-1)^. (c) photo of attaching position on exterior of SEM chamber.

# **Reference materials methods**

## **2.1 Raman**

Raman spectra were measured using a Renishaw inVia micro-Raman spectrometer with a 514 nm laser of 2 mW power and a 1 μm spot size.

Spectra are produced from a five sweep average, with cosmic ray removal. The step size was 0.97 cm^‑1^.

A representative Si wafer substrate on which CVD carbon specimens are deposited was also measured (**Figure S*2* (a)**).


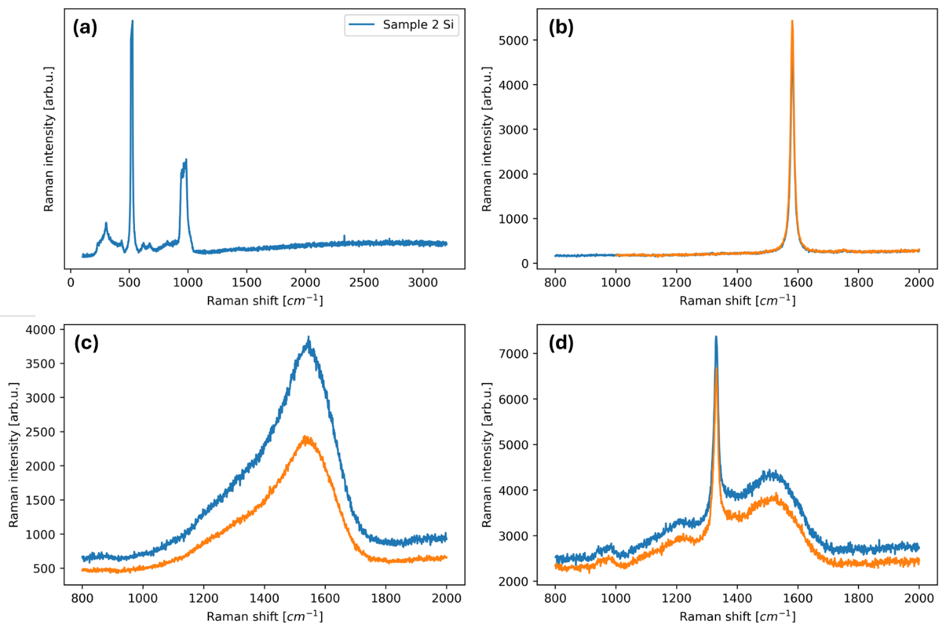


**Figure S2.** Raman spectra of (a) Si wafer substrate, and repeats of (b) HOPG, (c) CVD-1, (d) CVD-2.

## **XPS acquisition and analysis**

X-ray photoelectron spectroscopy (XPS) measurements were carried out in ultra-high vacuum using a Kratos AXIS Supra^+^ instrument. A monochromated aluminium X-ray source was operated at 75 W (15 kV anode voltage, 5 mA emission). The electrostatic and magnetic lens system was operated in the ‘hybrid’ mode with a ‘slot’ aperture defining an analysis area of approximately 700 μm × 300 μm on the samples. At least three analysis locations were measured on each sample. The incident X-rays were at an angle of 54.7^o^ (i.e. the ‘magic’ angle) to the analyser, and photoelectrons were measured at an emission angle of 0^o^ to the surface normal. Survey spectra were acquired using a pass energy of 80 eV, with two sweeps at a 0.5 eV step size, and ~90 ms dwell time. High-resolution narrow scans of the C 1s, O 1s, N 1s, and F 1s core levels were acquired using a pass energy of 20 eV, a 0.1 eV step size, and 200 ms dwell time. Repeat sweeps were acquired until either a signal-to-noise ratio of 200 or a maximum of 10 sweeps was reached. Spectra of the C KLL Auger peak were acquired using a pass energy of 40 eV, a step size of 0.5 eV, and 200 ms dwell time. Repeat sweeps were acquired until either a signal-to-noise ratio of 200 or a maximum of 50 sweeps was reached. Charge neutralization with a low-energy electron source was not required as none of the samples showed evidence of surface charging.

The analysis approach for C 1s peak fitting is detailed in [57]. Briefly, the C 1s high resolution spectrum from the HOPG sample was peak-ﬁtted using 4 components to obtain an empirical lineshape for the asymmetric graphitic component: two main HOPG components and two loss features (π‑π* transition and shake-up). The C 1s high resolution spectra from the DLC and CVD carbon samples were peak ﬁtted using the empirical HOPG lineshape and 6 additional components: non-graphitic C‑C, C‑O / C‑N, C=O / C‑F, O‑C=O, carbonates, and beta‑shifted O‑C=O.

## **SEHI**

### *Reference materials SE spectra collection*

Collection parameters for images in SEHI data volumes used to produce SE spectra of reference materials.

**Table S1** SEHI of reference materials collection parameters.

| parameter | unit / description | Values | | |
| --- | --- | --- | --- | --- |
|  |  | HOPG | DLC | CVD |
| BeamCurrent | pA | 12.50 | 12.50 | 12.50 |
| HV | kV | 1 | 1 | 1 |
| BeamMode | (U or N) | N-Beam | N-Beam | N-Beam |
| Dwell | ns | 50 | 50 | 50 |
| WorkingDistance | mm | 3.99 | 4.00 | 4.00 |
| StageT | rad | 0 | 0 | 0 |
| StageZ | mm | 3.99 | 4.00 | 4.00 |
| HorFieldsize | um | 10&20 | 10&20 | 10&20 |
| ResolutionX | pixels | 768 | 768 | 768 |
| ResolutionY | pixels | 512 | 512 | 512 |
| Average | frames | 16 | 16 | 16 |
| Integrate | frames | 0 | 0 | 0 |
| ScanInterlacing | lines | 8 | 8 | 8 |
| Contrast | % | 87 | 87 | 71.5 |
| Brightness | % | 41.3 | 41.3 | 41.3 |

SEHI data volumes were made of 49 image slices collected at mirror voltages 18 V to -5.04 V stepped by -0.48 V. Data volumes were acquired at 10 μm and 20 μm horizontal field widths (HFWs) at the same stage position (20 μm HFW SEHI data volume average image shown in **Figure S3(a)&(b)**). The spectra presented in the article **Figure 7(a)-(c)** were produced from the area in the 20 μm acquisition surrounding the 10 μm HFW (‘roi_outer’, **Figure S3(c) & (d))**.


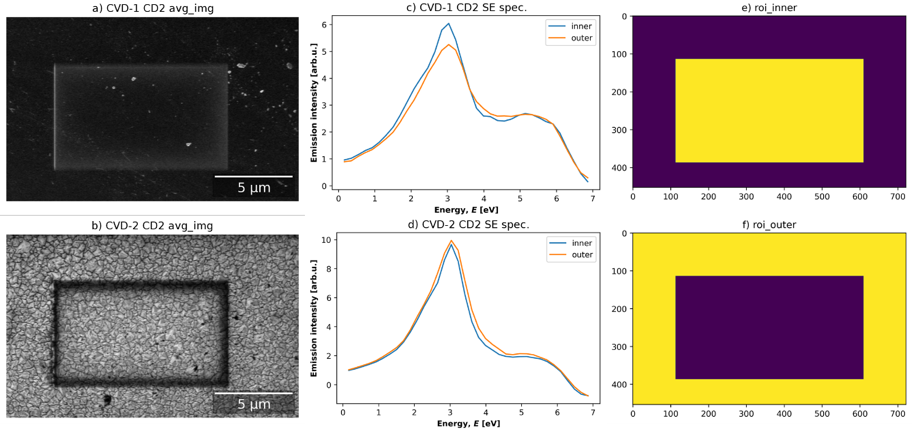


**Figure S3.** (a) & (b) 20 μm HFW SE images of CVD‑1 and CVD‑2 reference materials. (c) & (d) SE spectra from inner and outer regions. (e) & (f) Inner and outer regions where yellow indicates region included in the spectrum.

SE spectra from the ‘roi_outer’ region were averaged to produce the spectra in the reference material SE spectrum plots.


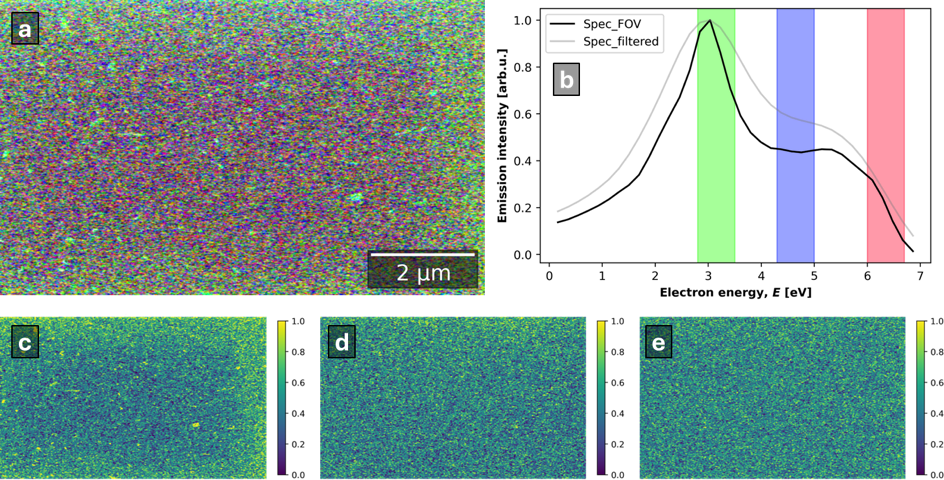


**Figure S4.** (a) RGB image of CVD-1 colored in energy ranges illustrated in (b) G: 2.8-3.5 eV for aCH, B: 4.3-5.0 eV for sp3 and R: 6.0-6.7 eV for sp2. (c)-(e) component maps for aCH, sp3 and sp2 respectively.


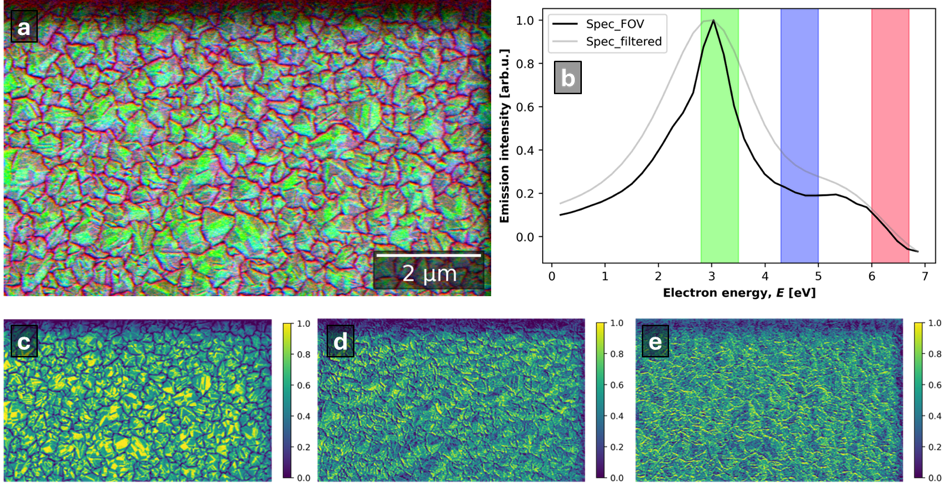


**Figure S5.** (a) RGB image of CVD-2 coloured in energy ranges illustrated in (b) G: 2.8-3.5 eV for aCH, B: 4.3-5.0 eV for sp3 and R: 6.0-6.7 eV for sp2. (c)-(e) component maps for aCH, sp3 and sp2 respectively.

### *2.3.2 Consideration of specimen charging*


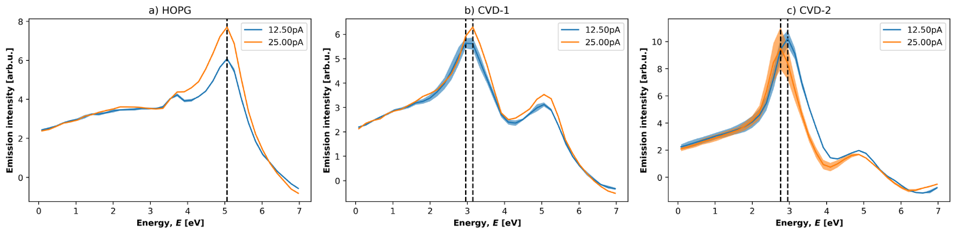


**Figure S6.** reference material plots from the outer region at 20 μm HFW at 12.50 pA and 25.00 pA beam currents measured by PFIB-SEM **D**. Peak center shifts versus 12.50 pA: (a) 0.00 eV, (b) +0.19 eV, (c) -0.19 eV.

### *2.3.3 Reference material SE spectrum fitting model initial parameters*

HOPG model initial parameters:

HOPG_model **=** **{**

'HOPG_1'**:{**'center' **:(**2**,**True**,**1.5**,**2.5**)},**

'HOPG_2'**:{**'center' **:(**5.5**,**True**,**5**,**6**)},**

'aCH' **:{**'center' **:(**3.99**,**True**,**3.5**,**4.5**)},**

'ebid' **:{**'center' **:(**1.53**,**True**,**1**,**2**)}**

**}**

Where the variables in the list are: center; vary; lower limit; upper limit.

Disordered carbon model for CVD carbons:

disordered_model **=** **{**

'HOPG_1'**:{**'center' **:(**2**,**True**,**1.5**,**2.5**),**

'amp' **:(**1**,**True**),**

'sigma' **:(**res_HOPG**.**values**[**'HOPG_1_sigma'**],**False**)},**

'HOPG_2'**:{**'center' **:(**5.5**,**True**,**5**,**6**),**

'amp' **:(**0.5**,**True**,**0.01**,**5**,**'3.1*HOPG_1_amplitude'**),**

'sigma' **:(**res_HOPG**.**values**[**'HOPG_2_sigma'**],**False**)},**

'aCH' **:{**'center' **:(**3.99**,**True**,**3**,**4.5**),**

'amp' **:(**1**,**True**)},**

'ebid' **:{**'center' **:(**1.53**,**True**,**1**,**2**),**

'amp' **:(**1**,**True**)},**

'sp3' **:{**'center' **:(**5**,** True**,** 4.8**,**5.2**),**

'amp' **:(**0.2**,**True**,**0.01**,**5**)}**

**}**

Where the variables in the list are: centre; vary; lower limit; upper limit; expression.

The sigma values are fixed to the result of the HOPG fit, and the amplitude of HOPG_2 is 3.1 times the amplitude of HOPG_1 (based on the ratio of HOPG_2:HOPG_1 amplitudes from the fit to the spectrum of HOPG).

### *2.3.4 Energy calibration*


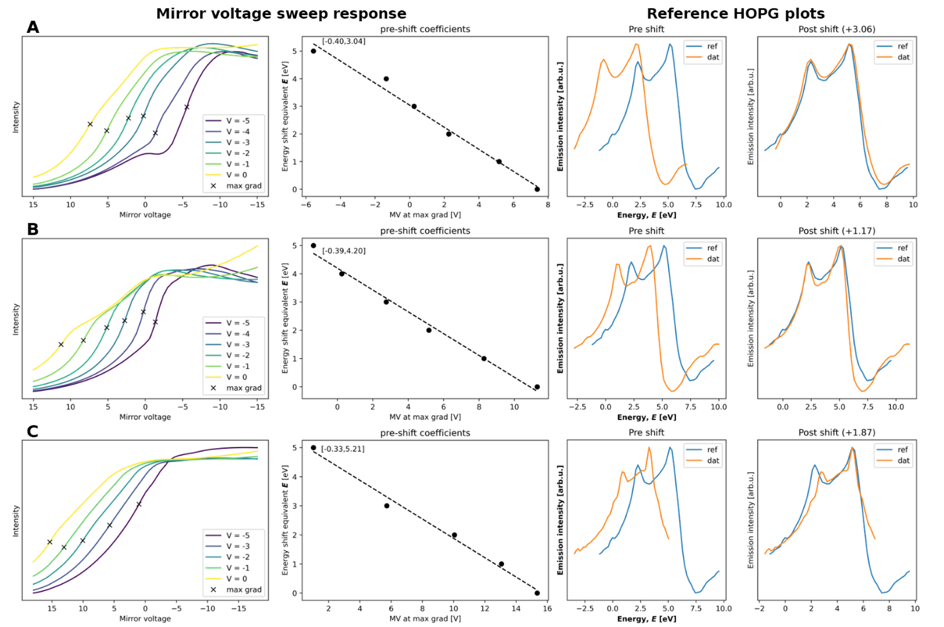


**Figure S7.** Energy filtering characteristics of five Elstar columns from the round-robin testing at five institutions from the stage biasing experiment. ‘S-curve’, energy calibration factor, energy shift and comparison to a reference spectrum of HOPG produced by (**A**) Helios NanoLab 660/G; (**B**) Helios G4 CX; (**C**) Helios G4 HP. ‘S-curve’ and CVD‑1 SE spectra plotted versus mirror voltage, electron energy from stage bias experiment.


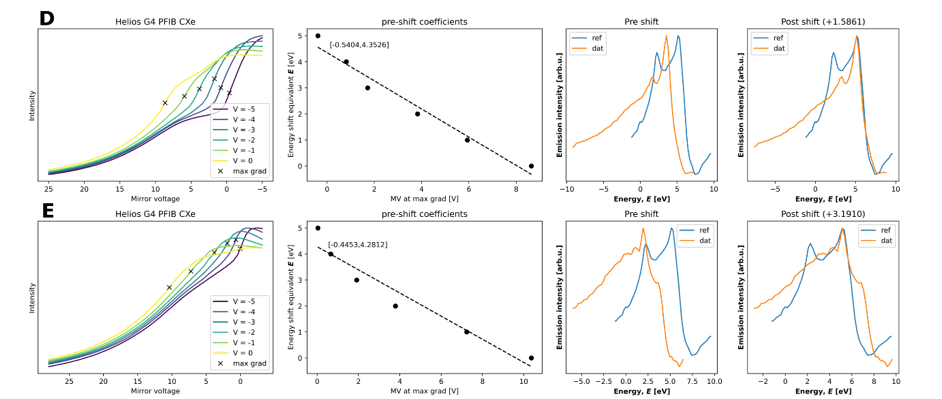


**Figure S8**. Stage biasing energy calibration for PFIB-SEMs **D** and **E**.


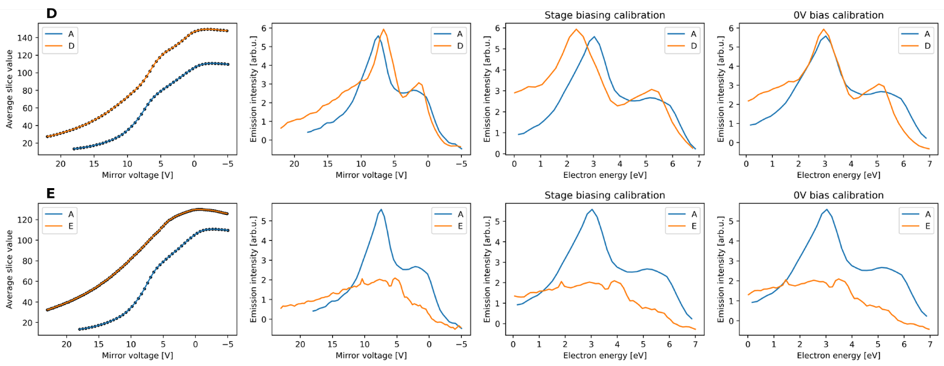


**Figure S9.** Calibration factor from (**A**) with shift for (**D**) Helios G4 PFIB CXe; (**E**) Helios G4 PFIB CXe.

## **2.4 Nano-FTIR characterization of EBID regions**

Atomic force microscopy (AFM) and nano Fourier transform infrared (nano‑FTIR) spectroscopy were used to investigate the character of an EBID surface produced during a SEHI measurement. AFM height mapping and nano-FTIR spectra were collected in ambient conditions using a neaSCOPE from Attocube Systems AG/Neaspec. The nano‑FTIR spectroscopy measurement sites are shown in the AFM height map region of 5 μm side length (**Figure S10(a)**). The AFM height map area (**Figure S10(c)**) shows the locations of nano‑FTIR absorbance spectra marked by crosses. 1a,b are located within the 26.89 Cm^-2^ dose region and 2a,b absorbance spectra are located in the 5.98 Cm^‑2^ dose region. Nano‑FTIR from two more regions, 3a,b, were from areas of HOPG not subject to electron beam radiation.

Absorbance peaks in the nano-FTIR spectra (**Figure S10(d)**) marked by dashed lines could be attributed as follows: 896 cm^‑1^ epoxide, 1057 cm^‑1^ ether, 1163 cm^‑1^ hydroxy [43], 1230 cm^‑1^ epoxide, 1472 cm^‑1^ aCH [4], 1480 cm^‑1^ amine [5], 1574-1630 cm^‑1^ graphite [43, 6, 4], 1700 cm^‑1^ carbonyl [43].

The alignment of peaks with these absorbance ranges from literature are telltale signatures of carbon and oxygen compounds on the surface of the HOPG. There is no trend between treated and untreated regions – the carbon and oxygen species are present in all areas and indicate contamination in air in all areas. Complex chemical signatures are difficult to identify and also we measure a layer 4.3 nm in height, which may not elicit a strong spectral response.


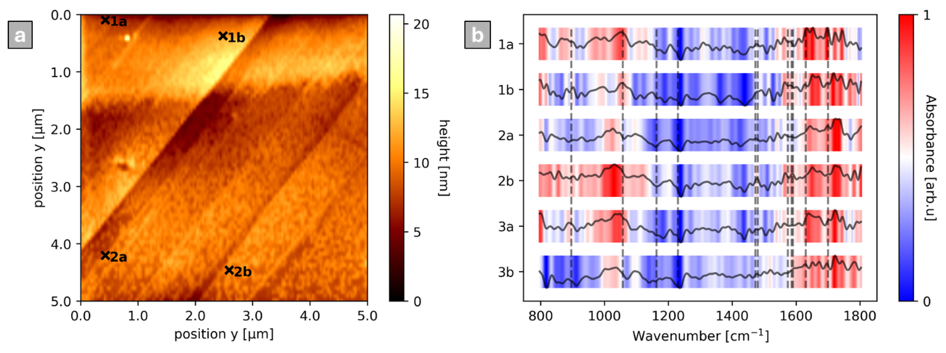


**Figure S10.** (a) AFM height map with nano-FTIR acquisition points marked. (b) Nano-FTIR spectra from the points shown in (a) where 1 denotes the ‘inner’ D_spec_ condition and 2 denotes ‘outer’ D_spec_ condition. 3a and 3b are nano-FTIR spectra from points where there was no electron dose.

## **In-situ SEHI measurement of EBID by cumulative electron dose experiment**

**Figure S11** shows the sequential SEHI experiment to evaluate EBID conditions.


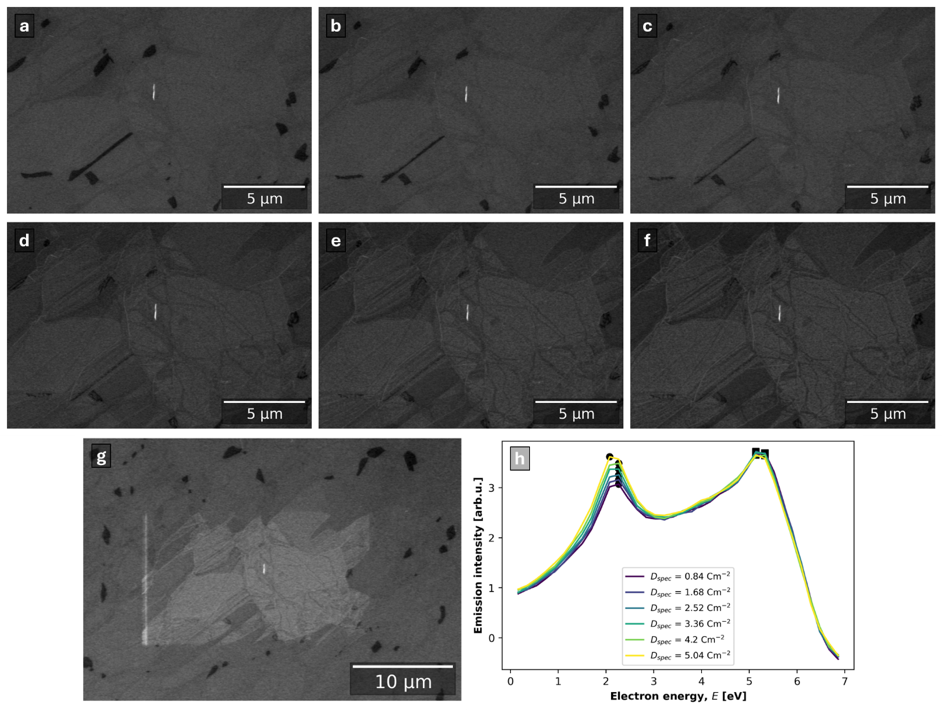


**Figure S11.** (a)-(f) SE images of 20 μm HFW produced from sequential SEHI measurements in the same area of HOPG. The electron doses received in (a)-(f) were 0.84 Cm^‑2^, 1.68 Cm^‑2^, 2.52 Cm^‑2^, 3.36 Cm^‑2^, 4.20 Cm^‑2^, 5.04 Cm^‑2^. Average chamber pressure was 1.02 mPa. (g) Overview SE image of the (a-f) area at 40 μm HFW. (h) SE spectra of HOPG from areas (a)-(f) with peak 1 maxima (circles) and peak 2 maxima (squares) marked.

The peak intensity values plotted in peak intensity versus spectrum dose plots are the maxima of the first and second peaks in the spectrum of HOPG. Note the chamber airlock equipped system does not have energy calibration, but peak intensity values can still be used for the purposes of the experiment as the spectrum shape is as expected for HOPG.


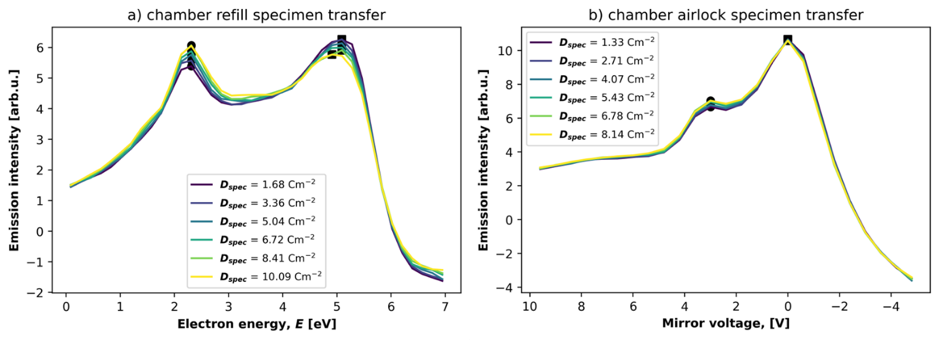


**Figure S12.** (a) sequential SEHI experiment SE spectra from the same region of HOPG surface.

# **Application materials methods**

## **3.1 Angular correction**

Angular correction was used to reduce shadowing effects from samples with high topography (graphite anode, LFP).
(a) stack acquired at stage rotation=0^o^,
(b) stack_r acquired at stage rotation=180^o^, (a) and (b) pixel values are scaled to 0-1 from uint8 datatype (0-255).
(c) half_diff=(stack-stack_r)/2,
(d) stack_AC=(stack-half_diff)*uint8.dtype_info.max.
Using data preprocessing package [available pysehi release].


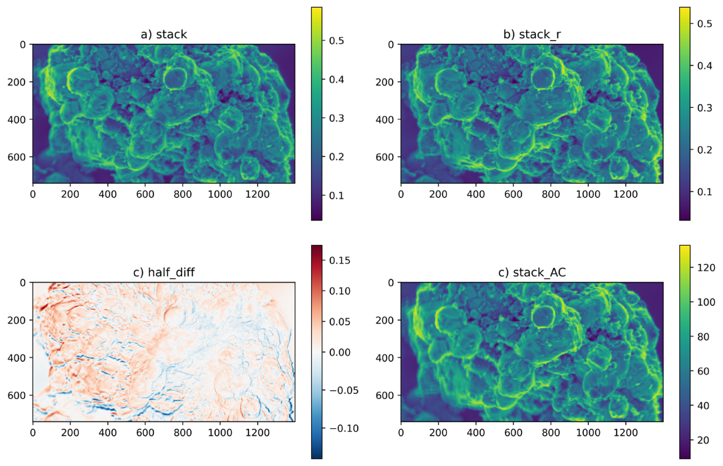


**Figure S13.** Angular correction acquisition and data pre-processing workflow.

## **3.2 Carbon coated lithium iron phosphate**


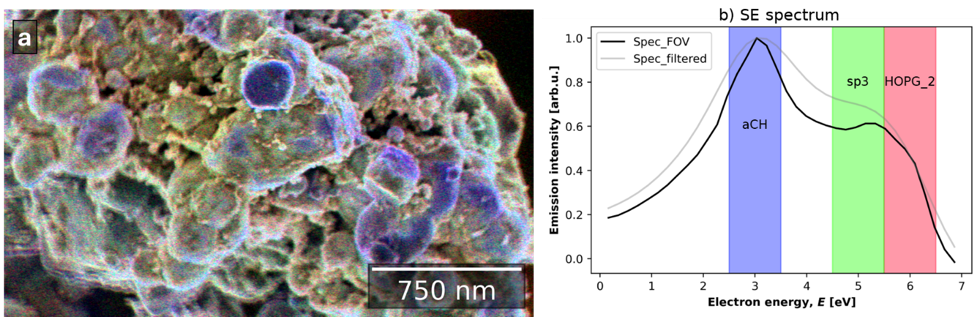


**Figure S14.** (a) CSEHI image using alternative colour assignment order to aid readers with difficulty distinguishing red-green colour contrast. (b) energy ranges used to make CSEHI image (a) are: HOPG_2:[5.5-6.5], sp3:[4.5-5.5], aCH:[2.5-3.5].


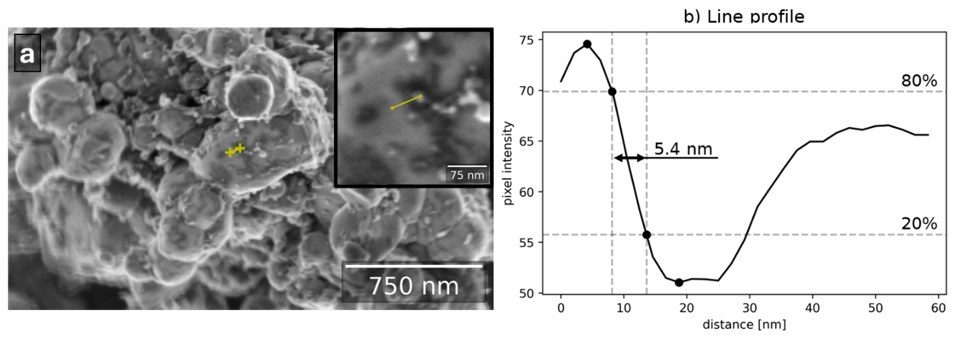


**Figure S15.** (a) LFP with line profile. (a, inset) magnified view of line profile region. (b) 20%-80% intensity measure of edge contrast, giving 5.4 nm distance between intensities over the edge.

Raman spectrum initial fit parameters model:

LFP_model **=** **{**

'PO4'**:** **{**'type'**:** 'lorentz'**,**

'center'**:** **(**950**,** True**,** 900**,**1000**),**

'amp'**:** **(**0.4**,**True**,** 0**,**100**),**

'sigma'**:** **(**5**,**True**,**0.001**,**10**)**

**},**

'aC'**:** **{**'type'**:** 'gauss'**,**

'center'**:** **(**1200**,**True**,**1100**,**1300**),**

'amp'**:** **(**0.5**,**True**,**0.001**,**50**),**

'sigma'**:** **(**1**,**True**,**0.001**,**100**)**

**},**

'Dis'**:** **{**'type'**:** 'gauss'**,**

'center'**:** **(**1380**,**True**,**1200**,**1450**),**

'amp'**:** **(**1**,**True**,**0.001**,**200**),**

'sigma'**:** **(**1**,**True**,**0.001**,**100**)**

**},**

'G'**:** **{**'type'**:** 'lorentz'**,**

'center'**:** **(**1550**,**True**,**1500**,**1700**),**

'amp'**:** **(**1**,**True**,**0.001**,**100**),**

'sigma'**:** **(**1**,**True**,**0.001**,**100**)**

**}**

**}**

## **3.3 Graphite electrode from a lithium ion battery**

The fitting model for the graphite active material region was the disordered carbon model as presented in the 0.

Initial fitting model for the binder domain:

binder_model **=** **{**

'HOPG_1'**:{**'center' **:(**HOPG_1_cen**,**False**),**

'amp' **:(**0.2**,**True**,**0.01**,**5**),**

'sigma' **:(**HOPG_1_sig**,**False**)},**

'HOPG_2'**:{**'center' **:(**HOPG_2_cen**,**False**),**

'amp' **:(**0.2**,**True**,**0.01**,**5**,**'3.1*HOPG_1_amplitude'**),**

'sigma' **:(**HOPG_2_sig**,**False**)},**

'aCH' **:{**'center' **:(**3.99**,**True**,**3**,**4.5**),**

'amp' **:(**1**,**True**,**0.01**,**5**),**

'sigma' **:(**1**,**True**,**0.1**,**3**)},**

'ebid' **:{**'center' **:(**1.53**,**True**,**1**,**2**),**

'amp' **:(**0.2**,**True**,**0.01**,**5**),**

'sigma' **:(**ebid_sig**,**False**)},**

'sp3' **:{**'center' **:(**5**,** True**,** 4.8**,**5.2**),**

'amp' **:(**0.2**,**True**,**0.01**,**5**),**

'sigma' **:(**1**,**True**,**0.1**,**2**)},**

'OH' **:{**'center' **:(**4.6**,** True**,** 4.2**,**5**),**

'amp' **:(**0.2**,**True**,**0.01**,**5**),**

'sigma' **:(**1**,**True**,**0.1**,**5**)},**

'CO' **:{**'center' **:(**5.2**,** True**,** 5**,**6.5**),**

'amp' **:(**0.2**,**True**,**0.01**,**5**),**

'sigma' **:(**1**,**True**,**0.1**,**2**)}**

**}**

Residual fit:


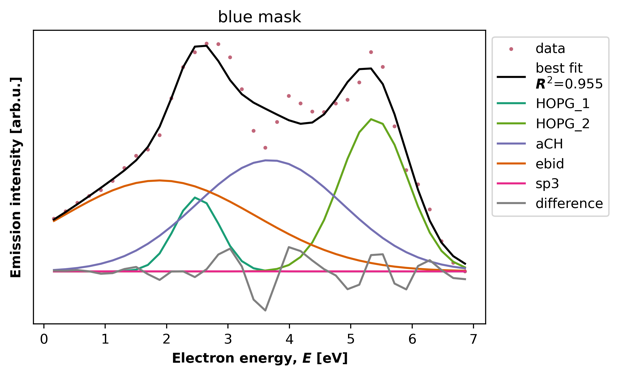


**Figure S16.** Blue mask region disordered carbon fit with residual.

## **3.4 CB characterization**

The preparation of ball-milled CBs is given in detail in the SI of [43]. The ball milling apparatus was the Pulverisette 7 Premium (Fritsch GmbH, Germany).

**Table S2** – Experimental conditions for ball milling. Reproduced from [43] under CC BY 4.0 license^[[2]](#footnote-2)^.

| ball-to-powder weight ratio | 150 |
| --- | --- |
| rotation frequency [min−1] | 300, 500, 700 |
| ball size [mm] | 10 |
| total balls weight [g] | 15 |
| milling tool material | Silicon nitride |
| beaker volume [cm3] | 80 |
| CB weight [mg] | 100 |
| sample notation | oCB-1h, oCB-5h, oCB-9h, oCB-11h, oCB-13h |

### *3.4.1. CB SE spectrum fit*

The initial fit parameters and constraints for fitting SE spectra of the CB:

CB_model **=** **{**

'HOPG_1'**:{**'center' **:(**HOPG_1_cen**,**True**,**1.5**,**2.5**),**

'amp' **:(**0.2**,**True**,**0.01**,**5**),**

'sigma' **:(**HOPG_1_sig**,**False**)},**

'HOPG_2'**:{**'center' **:(**HOPG_2_cen**,**True**,**5**,**6**),**

'amp' **:(**0.2**,**True**,**0.01**,**5**,**'3.1*HOPG_1_amplitude'**),**

'sigma' **:(**HOPG_2_sig**,**False**)},**

'aCH' **:{**'center' **:(**3.99**,**True**,**3**,**4.5**),**

'amp' **:(**1**,**True**,**0.01**,**5**),**

'sigma' **:(**1**,**True**,**0.1**,**5**)},**

'ebid' **:{**'center' **:(**1.53**,**True**,**1**,**2**),**

'amp' **:(**0.2**,**True**,**0.01**,**5**),**

'sigma' **:(**ebid_sig**,**False**)},**

'sp3' **:{**'center' **:(**5**,** True**,** 4.8**,**5.2**),**

'amp' **:(**0.2**,**True**,**0.01**,**5**)},**

'OH' **:{**'center' **:(**4.6**,** True**,** 4.4**,**4.8**),**

'amp' **:(**0.2**,**True**,**0.01**,**5**)},**

'CO' **:{**'center' **:(**5.2**,** True**,** 5**,**5.4**),**

'amp' **:(**0.2**,**True**,**0.01**,**5**)}**

**}**

Where the variables in the list are: center; vary; lower limit; upper limit; expression.

The sigma values are fixed to the result of the HOPG fit, and the amplitude of HOPG_2 is 3.1 times the amplitude of HOPG_1 (based on the ratio of HOPG_2:HOPG_1 amplitudes from the fit to the spectrum of HOPG).

### *3.4.2. CB line profile region*


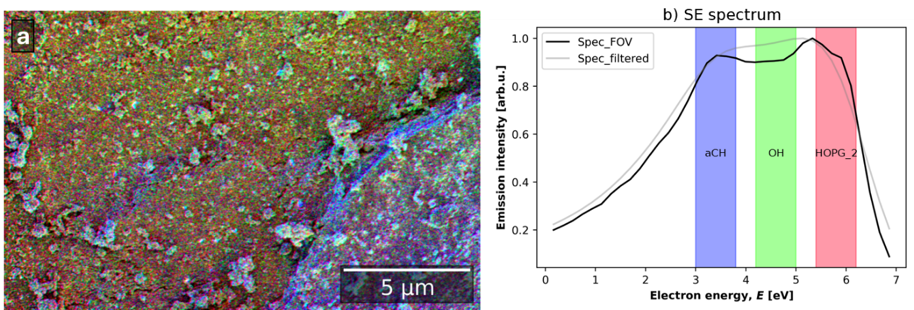


**Figure S17.** (a) CSEHI image using alternative color assignment order to aid readers with difficulty distinguishing red-green color contrast. (b) energy ranges used to make CSEHI image (a) are: HOPG_2:[5.4-6.2], OH:[4.2-5.0], aCH:[3.0-3.8].


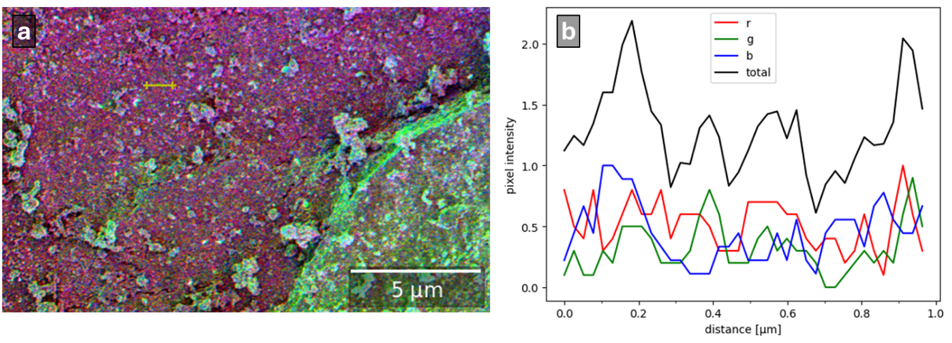


**Figure S18**. (a) CSEHI image produced with ranges R:‘HOPG_2’, [5.4‑6.2 eV]; G:‘aCH’, [3‑3.8 eV]; B:‘OH’, [4.2‑5.0 eV]. 1 μm line profile plotted on image. (b) plot of pixel intensities in line profile region.

# **References**

[1] A. Suri, A. Pratt, S. Tear, C. Walker, and M. El-Gomati, “Next generation secondary electron detector with energy analysis capability for SEM,” *Journal of Microscopy*, vol. 279, no. 3, pp. 207–211, Feb. 2020.

[2] S. Marchesini, P. Turner, K. R. Paton, B. P. Reed, B. Brennan, K. Koziol, and A. J. Pollard, “Gas physisorption measurements as a quality control tool for the properties of graphene/graphite powders,” *Carbon*, vol. 167, pp. 585–595, Oct. 2020.

[3] A. Kiani, M. R. Acocella, V. Granata, E. Mazzotta, C. Malitesta, and G. Guerra, “Green oxidation of carbon black by dry ball milling,” *ACS Sustainable Chemistry &amp Engineering*, vol. 10, no. 48, pp. 16019–16026, nov 2022.

[4] T. Leitner, J. Kattner, and H. Hoffmann, “Infrared reflection spectroscopy of thin films on highly oriented pyrolytic graphite,” *Applied Spectroscopy*, vol. 57, no. 12, pp. 1502–1509, Dec. 2003.

[5] J Przepiórski, M Skrodzewicz, and A.W Morawski. High temperature ammonia treatment of activated carbon for enhancement of CO_2_ adsorption. *Applied Surface Science*, 225(1-4):235–242, March 2004.

[6] C.D. Zappielo, D.M. Nanicuacua, W.N.L. dos Santos, D.L.F. da Silva, L.H. Dall’Antônia, F.M. de Oliveira, D.N. Clausen, and CR.T. Tarley. Solid phase extraction to on-line preconcentrate trace cadmium using chemically modified nano-carbon black with 3-mercaptopropyltrimethoxysilane. *Journal of the Brazilian Chemical Society*, 2016.

1. https://creativecommons.org/licenses/by/4.0/ [↑](#footnote-ref-1)
2. https://creativecommons.org/licenses/by/4.0/ [↑](#footnote-ref-2)
